# Molecular fingerprints are strong models for peptide function prediction

**DOI:** 10.1093/bioinformatics/btag179

**Published:** 2026-04-13

**Authors:** Jakub Adamczyk, Piotr Ludynia, Wojciech Czech

**Affiliations:** Faculty of Computer Science, AGH University of Krakow, Krakow, 30-059, Poland; Faculty of Computer Science, AGH University of Krakow, Krakow, 30-059, Poland; Faculty of Computer Science, AGH University of Krakow, Krakow, 30-059, Poland

## Abstract

**Motivation:**

Understanding peptide properties is often assumed to require modeling long-range molecular interactions, motivating complex graph neural networks and pretrained transformers. Whether such long-range dependencies are essential remains unclear. We investigate if simple, domain-specific molecular fingerprints can capture peptide function without these assumptions. Atomic-level representations aim to provide richer information than purely sequence-based models and better efficiency than structural ones.

**Results:**

Across 132 datasets, including LRGB and five additional peptide benchmarks, models using count-based ECFP, Topological Torsion, and RDKit fingerprints with LightGBM achieve state-of-the-art accuracy. Despite encoding only short-range molecular features, these models outperform GNNs and transformer-based approaches. Control experiments confirm that fingerprints, though inherently local, suffice for robust peptide property prediction. Our results challenge the presumed necessity of long-range interaction modeling and highlight molecular fingerprints as efficient, interpretable, and lightweight alternatives.

**Supplementary information:**

All code and data are available on GitHub and Zenodo: https://github.com/scikit-fingerprints/peptides_molecular_fingerprints_classification  https://doi.org/10.5281/zenodo.19388783

## Introduction

Peptides are short chains of amino acids, typically composed of 3–50 residues, that perform diverse biological functions and are increasingly being studied as potential therapeutics. They can bind to proteins, regulate their activity, and show antiviral, antitoxin, anticancer, and antidiabetic properties (Wang et al. 2022, Goles et al. 2024). In particular, antimicrobial peptides (AMPs) are promising candidates against the growing antimicrobial resistance crisis (Szymczak et al. 2023). Therefore, accurate prediction of peptide properties is essential for drug discovery.

Peptide function prediction poses unique challenges for machine learning (ML), including homology bias and imbalanced datasets (Sidorczuk et al. 2022, Fernández-Díaz et al. 2024). Consequently, numerous benchmark datasets with standardized evaluation procedures have been created. Peptides can be represented through various modalities, influencing the choice of ML models. When 3D structures are available, structural encodings can be used (Spänig et al. 2021), but these are rare and computationally expensive to obtain. Thus, sequence- and graph-based representations that avoid folding simulations have gained more attention.

Recent years have seen a shift from handcrafted sequence features to pretrained protein language models (PLMs) such as ProtBERT and ESM (Elnaggar et al. 2022, Lin et al. 2023). Alternatively, peptides can be modeled as molecular graphs, enabling the use of Graph Neural Networks (GNNs) and Graph Transformers (GTs). The Long-Range Graph Benchmark (LRGB) (Dwivedi et al. 2022) formulated peptide prediction as a long-range dependency problem. However, the practical importance of long-range interactions in short and flexible peptides remains unclear.

In chemoinformatics, molecular fingerprints—compact encodings derived from small molecular subgraphs (Todeschini and Consonni 2009) - are widely used for prediction of small-molecule properties. They offer domain-specific, efficient representations that often rival deep learning methods (Jiang et al. 2021, Xia et al. 2023). Despite their success, their use for peptide prediction has been limited, with only a few hybrid approaches that incorporate binary fingerprints (Kang et al. 2024, Tan et al. 2024).

In this work, we revisit count-based molecular fingerprints as features for peptide function prediction. When combined with a LightGBM classifier, these representations achieve state-of-the-art results across six benchmarks, including LRGB, without requiring hyperparameter tuning or 3D structural information. This suggests that short-range subgraph statistics may be sufficient to capture key biochemical features, challenging the assumption that long-range dependencies are essential.

Our contributions are threefold, as we (1) demonstrate that count-based molecular fingerprints outperform alternative models on 132 datasets, (2) provide the most comprehensive benchmark of fingerprint-based peptide models to date, and (3) we analyze the proposed method in controlled experiments, showing its robustness.

## Related works

Prediction approaches for peptides differ mainly by representation. For atom-level graphs, message-passing GNNs such as GCN, GraphSAGE, and GIN rely on local neighborhood aggregation (Hamilton et al. 2017, Kipf and Welling 2017, Xu et al. 2019). Graph Transformers replace message passing with attention mechanisms, typically with sparsified or semi-local variants, rather than fully global attention, to manage computational cost. Their rchitectures, like GraphGPS, HDSE, GRIT, GraphViT, and S^2^GCN, aim to combine local and long-range modeling, and report strong performance on peptides datasets in LRGB benchmark (Rampášek et al. 2022, He et al. 2023, Ma et al. 2023, Geisler et al. 2024, Luo et al. 2024).

Sequence-based pipelines remain competitive, especially when data is scarce. They derive physicochemical and compositional descriptors (e.g. amphiphilicity, hydrophobicity) and use classical classifiers like RandomForest or SVM. Notable examples include Ampir, MACREL and amPEPpy (Santos-Júnior et al. 2020, Fingerhut et al. 2021, Lawrence et al. 2021), and early deep learning models such as AMPScannerV2 (Veltri et al. 2018). More recently Protein Language Models (PLMs), based on transformer architecture, such as ProtBERT and ProtT5 gained popularity, utilizing pretraining on large protein corpora (Elnaggar et al. 2022). The ESM family of models shows a scaling behavior similar to LLMs and give strong results on independent peptide benchmarks (Lin et al. 2023, Fernández-Díaz et al. 2024). For AMPs, specialized PLMs are available, e.g., BERT-Protein, LMPred, cAMPs-pred, and AMP-BERT (Zhang et al. 2021, Dee 2022, Ma et al. 2022, Lee et al. 2023).

Our work differs in adopting domain-specific, count-based molecular fingerprints as peptide structure encoders. Compared with deep models, they provide robust, low-parameter features and, as we show, give strong performance in peptide function prediction tasks without incorporating long-range mechanisms.

## Methods

Here, we describe the molecular fingerprints that form our proposed atom-level peptide encoding. We also describe the approach of Long Range Graph Benchmark to testing the presence of long-range dependencies.

### Molecular fingerprints

**Figure 1:**
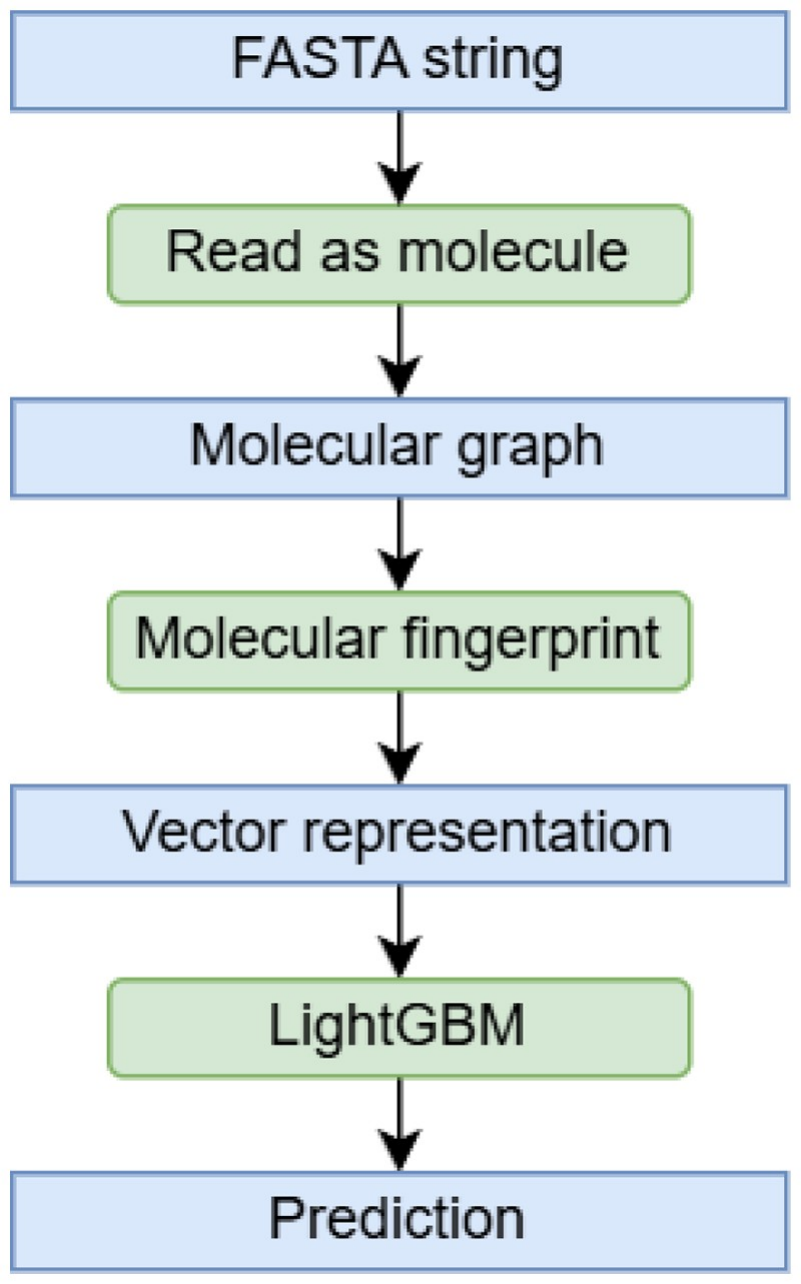
The proposed pipeline for peptide function prediction.

Molecular fingerprints are feature extraction algorithms for molecules, based on extracting small subgraphs and detecting their presence (binary variant) or counting occurrences (count variant). This turns the problem of molecular graph classification into tabular classification, typically combined with tree-based ensembles such as Random Forest or LightGBM (Ke et al. 2017). See Figure 1 for visualization of the proposed pipeline.

We focus on hashed fingerprints (Todeschini and Consonni 2009), exemplified by ECFP (Rogers and Hahn 2010) or Topological Torsion (Nilakantan et al. 1987). They are more flexible than structural fingerprints like MACCS or PubChem, which use predefined subgraphs like functional groups, and are not designed for peptides. Hashed fingerprints use a more flexible approach, defining a general “shape” of extracted subgraphs, for example, circular atom neighborhoods in ECFP (Rogers and Hahn 2010), paths of given length in Topological Torsion (TT) (Nilakantan et al. 1987), or all small subgraphs in RDKit fingerprint (Landrum et al. 2025). Extracting subgraph features in this manner avoids predefining features explicitly, offering greater flexibility. Subgraphs are defined by their structure, including the specific atoms and bonds they contain (e.g. characterized by atomic numbers and bond types), which are then converted into unique integer identifiers. To obtain a constant-length representation, they are hashed into the output vector, typically using the modulo function. The binary variant only indicates whether a given substructure appears in a molecule at all, while the count-based variant tallies the occurrences, incorporating more information about the compound composition and size.

**Figure 2:**
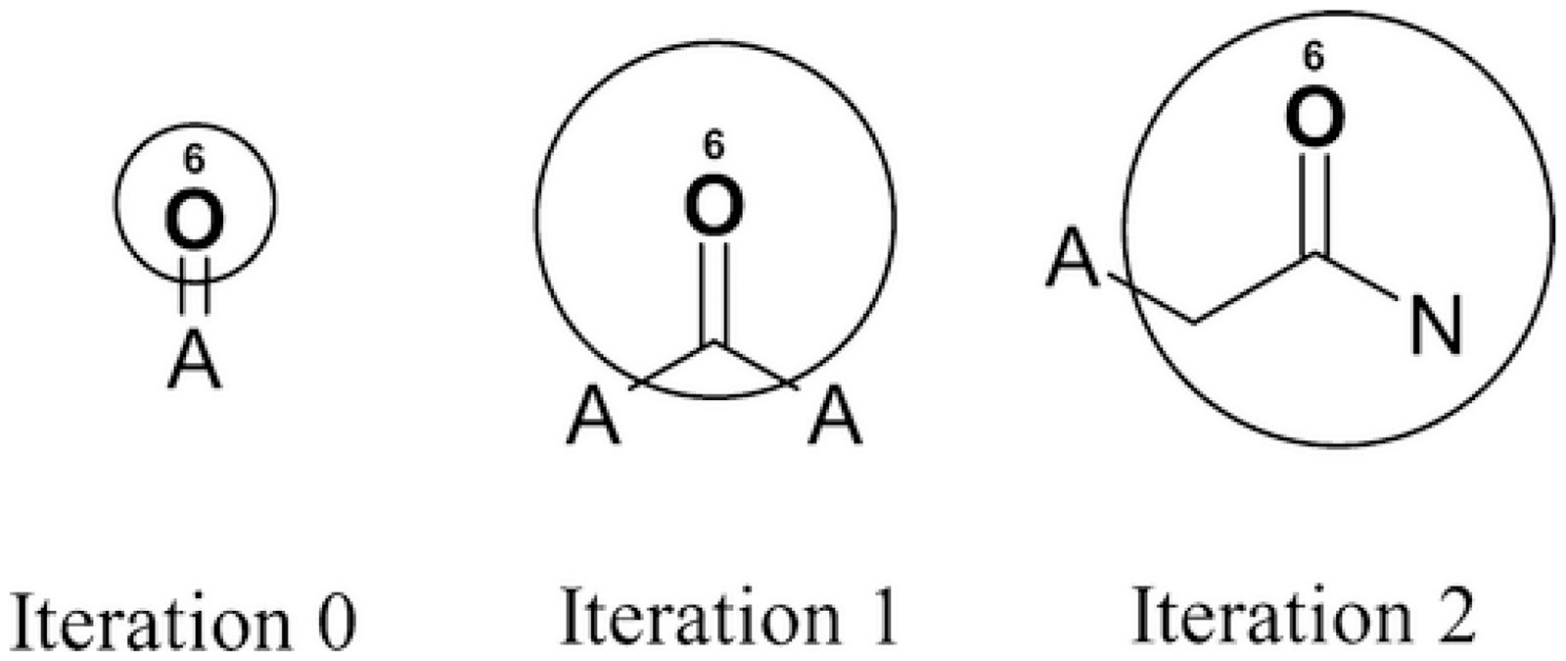
Example subgraph extraction in consecutive iterations of ECFP algorithm. Reprinted with permission from (Rogers and Hahn 2010). Copyright 2010 American Chemical Society.

ECFP typically uses circular subgraphs of radius 2 (see Figure 2). It starts with the atom itself, creating a numerical identifier based on atomic number, number of heavy neighbors, valence, and a few other simple features (see (Rogers and Hahn 2010) for a complete list). Then, it iteratively increases the radius, incorporating neighbors by combining their identifiers, up to a given maximal radius. This results in the final subgraph identifier for each atom. Such a procedure, known as the Morgan algorithm, closely resembles the operation of message passing in GNNs, as both are rooted in the Weisfeiler-Lehman isomorphism test. Topological Torsion (Nilakantan et al. 1987), designed to model short-range molecular interactions, encodes linear paths of length 4, combining the identifiers of these consecutive atoms. RDKit fingerprint (Landrum et al. 2025) uses all subgraphs of size up to 7 bonds, which can be nonlinear and include cycles (e.g. rings). The major difference between TT and RDKit is that the former uses only linear paths, while the RDKit fingerprint also encodes small cyclic structures.

The shapes of subgraphs extracted by ECFP with radius 2 are roughly equivalent to those of a shallow 2-layer GNN (Duvenaud et al. 2015). However, fingerprints do not learn task-specific weights like GNNs, instead performing domain-specific, deterministic feature extraction. This naturally reduces their risk of overfitting and increases the robustness of the learned feature space (Jiang et al. 2021, Xia et al. 2023), particularly beneficial in small, noisy datasets typical in chemo- and bioinformatics.

We focus on the three aforementioned fingerprints, as they are strictly local, very short-range descriptors. Therefore, a good performance of models based on their features can be attributed only to the importance of the short-range relations, rather than to long-range dependencies, since they are inherently incapable of capturing them. This locality makes them notably fast to compute (Adamczyk and Ludynia 2024) and scalable to larger molecules like peptides, e.g. ECFP scales linearly with the number of atoms. Because peptides are relatively linear structures, path-based TT and RDKit fingerprints also scale well. Those three fingerprints have also shown remarkable performance in ligand-based virtual screening (Riniker and Landrum 2013).

**Figure 3:**
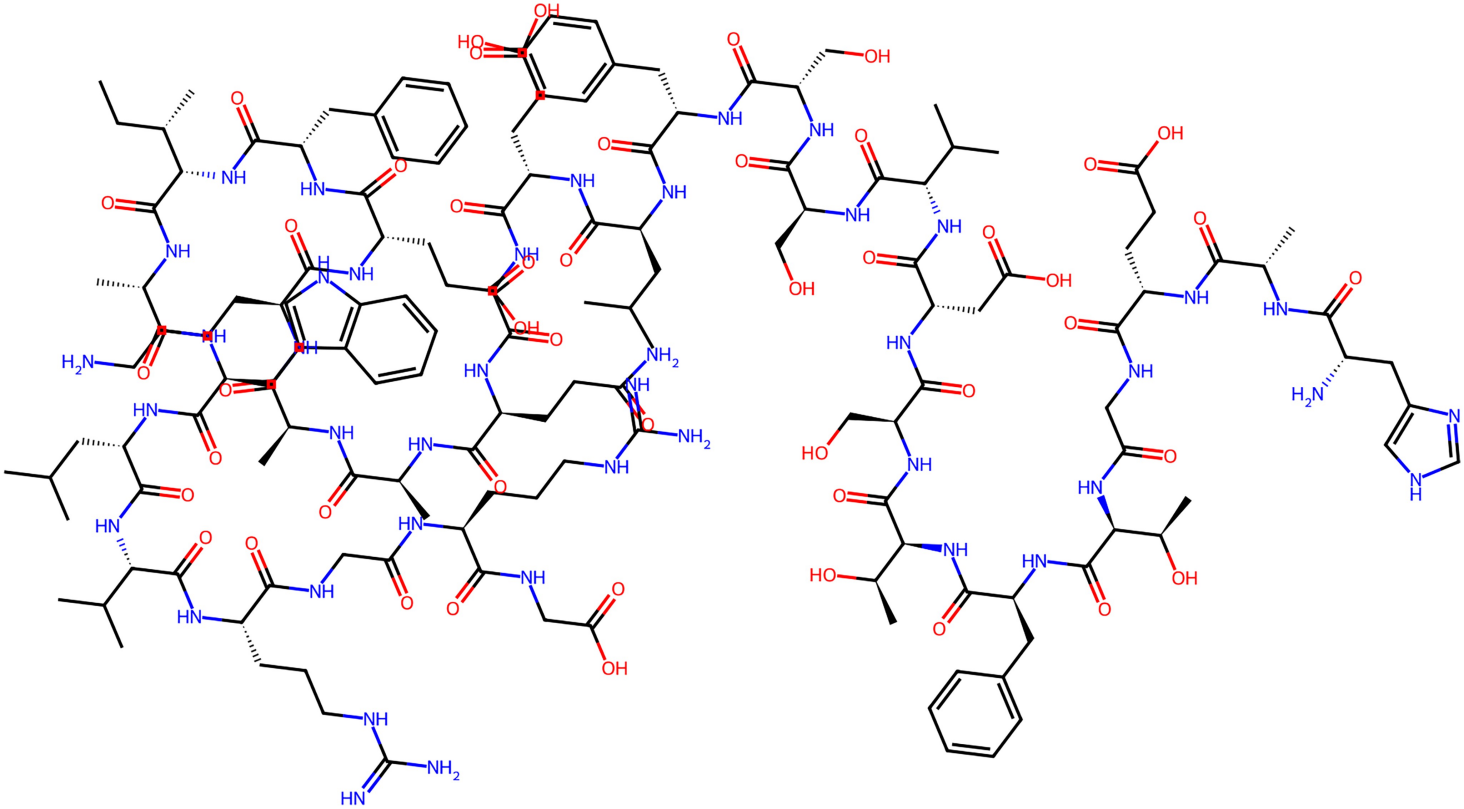
Atom-level molecular graph for Liraglutide peptide.

Lastly, we note that molecular fingerprints do not require any information about molecular conformation, peptide folding, or even whether a stable structure exists at all. Atom-level graphs (see Figure 3 for an example) can be quickly and deterministically constructed from an amino acid sequence, and then vectorized by a fingerprint algorithm.

### Long-range interactions (LRI)

Long Range Graph Benchmark (LRGB) (Dwivedi et al. 2022) proposed 3 conditions for a dataset to potentially require learning long-range interactions (LRI). Firstly, a necessary condition is that the graphs must be sufficiently large, significantly exceeding the typical GNN receptive field. Secondly, the nature of the task itself must require long-range dependencies between nodes, with local interactions being insufficient. Lastly, the experiments should confirm this—models with global knowledge, such as GNNs with topological encodings or graph transformers, must outperform local message-passing ones. For graph classification and regression, peptide property prediction was proposed as a task family meeting these conditions.

We show that this is not necessarily the case, especially for the third condition. While atomic-level peptide graphs are large and elongated (large diameter, low atom degrees) enough to suggest potential LRI, the biological motivation for such interactions in peptide property prediction is weaker than for larger proteins. In proteins, LRI arise from complex folding driven by distant amino acid interactions and strongly influence functional properties. By contrast, the smaller size of peptides naturally limits these effects and often leads to a lack of well-defined structure (Iglesias et al. 2025).

The third point, namely the superior performance of models with global knowledge, is the easiest to validate quantitatively. Since the argument is based on experimental results, it suffices to show that inherently local short-range models outperform long-range ones. Molecular fingerprints exactly fulfill these requirements. By obtaining SOTA results, we show that they provide strong evidence against the third claim, indicating that peptide function prediction primarily depends on short-range interactions.

## Experiments and results

We train the proposed fingerprint-based models on a diverse set of benchmarks covering a wide range of peptide properties, data sampling strategies, evaluation metrics, and train-test splits. We closely follow the procedures of the original benchmark publications and report the corresponding metrics. When multiple metrics are available, we report AUROC and Matthews Correlation Coefficient (MCC) due to space constraints, with the remaining metrics provided in the Supplementary Material. The best result in each table is highlighted in bold.

Our proposed fingerprint-based models use count variants throughout and are implemented with the scikit-fingerprints (Adamczyk and Ludynia 2024) library. We use default values for all hyperparameters: ECFP radius 2 (diameter 4, i.e., ECFP4), Topological Torsion path length 4, and RDKit fingerprint path length 7. They use small subgraphs as features, capturing only very short-range dependencies. We observed no significant performance gains from hyperparameter tuning (except for PeptideReactor, see Section General peptide benchmarks).

We use LightGBM (Ke et al. 2017) with 500 trees as the classifier due to its robust performance. Since most datasets are highly imbalanced, we apply class weighting inversely proportional to the positive class frequency. All other hyperparameters are kept at their default values, as tuning yielded negligible improvements. Increasing the number of trees beyond the default 100 was the only change that consistently improved performance, in line with prior literature (Probst et al. 2019).

The proposed method’s low sensitivity to hyperparameters is advantageous, as it reduces computational cost for new tasks, especially in large-scale virtual screening of peptide therapeutics. It also provides a fast and easy-to-use baseline for future works in this area.

### Long range graph benchmark (LRGB)

We first report results on the LRGB (Dwivedi et al. 2022) benchmark for both datasets—*Peptides-func* (classification) and *Peptides-struct* (regression)—in Table 1. These are multioutput datasets, and we report metrics averaged across all tasks. We compare against a broad set of graph-based architectures, including the original Graph Transformer and SAN (Dwivedi et al. 2022), as well as more recent models such as the MOLTOP baseline (Adamczyk and Czech 2024), well-tuned classical GNNs (GCN, GatedGCN, GINE) (Tönshoff et al. 2024), and models with enhanced long-range capabilities including GRIT (Ma et al. 2023), HDSE (Luo et al. 2024), and S^2^GCN (Geisler et al. 2024).

Since both datasets are multitask and LightGBM supports only single outputs, we train a separate model per task. For *Peptides-struct*, we optimize MAE loss function. LRGB results for GNNs are reported over 10 random seeds. LightGBM with default settings is deterministic, so we do not report standard deviations. Results for Random Forest and Extremely Randomized Trees, which depend on random seed, are provided in the Supplementary Material. Their standard deviations are very low, <0.1 AUPRC.

The key observation is that all fingerprint-based models achieve SOTA results on both datasets. The strictly local ECFP model, based on radius 2 subgraphs, exceeds the best long-range GNN, S^2^GCN, by 1.5% AUPRC. Topological Torsion and RDKit fingerprint obtain similar results. The fact that these inherently short-range models reach such a performance contradicts the conclusions of (Dwivedi et al. 2022) that incorporating long-range dependencies is necessary for accurate peptide function prediction. Instead, our results underscore the dominant role of short-range interactions.

The ECFP4 fingerprint functions similarly to a two-layer message-passing GNN, yet it outperforms both local models (e.g., GCN) and long-range variants such as S^2^GCN and HDSE. We attribute this to three factors.

First, fingerprints count discrete substructures rather than learning continuous graph representations. This provides a stronger inductive bias and reduces reliance on large training datasets.

Second, peptides consist of repeating fragments, amino acids, that are structurally similar and recur frequently. Thus, models mainly need to detect recurring small subgraphs (e.g., amine groups, -NH2). This is not indicative of genuine long-range interaction, but rather reflects the straightforward repetition of common motifs across different regions of the input.

Third, count-based fingerprints naturally capture effects of structure size and substructure frequency. In *Peptides-struct*, regression targets such as inertia mass and length strongly correlate with peptide size. In *Peptides-func*, classification labels (e.g., antimicrobial activity) depend on binding properties linked to surface area and thus size. These properties are therefore more closely tied to local structures and overall peptide size than to long-range interactions, and can be well approximated by counting subgraphs.

Our results therefore indicate that the *Peptides-func* and *Peptides-struct* datasets do not require modeling long-range interactions. Previous studies may have overlooked this, as (Dwivedi et al. 2022) evaluated only GNNs, and (Tönshoff et al. 2024) focused on deep models (6 to 10 layers) with global topological encodings. This underscores the importance of comparing against established, domain-specific baselines for a fair evaluation. To emphasize the effect of count-based fingerprints, we compare them with (1) binary variants (2) binary variants with sequence length feature, see Table 2. We observe substantial performance gains from using count features instead of binary ones. They also strongly outperform binary fingerprints with sequence length, meaning that the count-based model does not simply approximate the overall peptide size. Given those findings, it is concerning that all hybrid models in the bioinformatics literature we reviewed (Kang et al. 2024, Tan et al. 2024) rely exclusively on binary fingerprints, despite count variants having identical computational cost and markedly better performance.

**Table 1:** The results on LRGB benchmark.

Model	Peptides-func AUPRC↑	Peptides-struct MAE ↓
Transformer	63.26 ± 1.26	0.2529 ± 0.0016
SAN	64.39 ± 0.75	0.2545 ± 0.0012
MOLTOP	64.59 ± 0.05	–
GraphGPS	65.35 ± 0.41	0.2500 ± 0.0005
GINE	66.21 ± 0.67	0.2473 ± 0.0017
GatedGCN	67.65 ± 0.47	0.2477 ± 0.0009
GCN	68.60 ± 0.50	0.2460 ± 0.0007
GraphViT	69.42 ± 0.75	0.2449 ± 0.0016
GRIT	69.88 ± 0.82	0.2460 ± 0.0012
CRaWl	70.74 ± 0.32	0.2506 ± 0.0022
GRED	71.33 ± 0.11	0.2455 ± 0.0013
DRew	71.50 ± 0.44	0.2536 ± 0.0015
HDSE	71.56 ± 0.58	0.2457 ± 0.0013
S^2^GCN	73.11 ± 0.66	0.2447 ± 0.0032
RDKit	73.11	0.2459
TT	73.18	0.2438
**ECFP**	**74.60**	**0.2432**

**Table 2:** Differences between binary and count fingerprint models.

Dataset	Variant	ECFP	TT	RDKit
Peptides-func	binary	70.57	66.18	63.88
Peptides-func	binary + length	68.46	66.49	66.97
Peptides-func	count	74.60	73.18	73.11
AUPRC gain ↑		+4.03	+7.00	+9.23
Peptides-struct	binary	0.3049	0.3298	0.3331
Peptides-struct	binary + length	0.2652	0.2691	0.2744
Peptides-struct	count	0.2432	0.2438	0.2459
MAE gain ↓		−0.0617	−0.0860	−0.0872

We also highlight the efficiency of our approach. In terms of wall-clock time, computing ECFP fingerprints and training LightGBM takes 19 seconds on a 12-core Intel Core i7-12700KF CPU for *Peptides-func*. In contrast, training the SAN graph transformer requires up to 60 hours on an NVIDIA A100 GPU (Dwivedi et al. 2022). Additional timing results are provided in the Supplementary Material.

### Antimicrobial peptides (AMPs) benchmarks

In subsequent experiments, we examine whether the LRGB results generalize to other benchmarks in this domain. Such generalization would indicate that peptide function prediction is not inherently dependent on long-range interactions.

We first report results on antimicrobial peptide (AMP) prediction benchmarks. Although those benchmarks target the same property, antimicrobial activity, they differ in structural diversity, construction of positive and negative examples, and train-test splitting procedures. We consider three benchmarks that span these variations: the BERT-based models benchmark (Gao et al. 2024), XUAMP (Xu et al. 2021), and AMPBenchmark (Sidorczuk et al. 2022). Notably, many datasets in this section include peptides that exceed the typical length definition, with averages of 70–100 amino acids. Achieving strong performance therefore requires fingerprint-based models to be both scalable, due to atom-level processing, and effective on substantially larger molecules. We include detailed dataset descriptions and sequence length statistics in Supplementary Material.

#### BERT-based models benchmark

First, we compare our approach with BERT-based models pretrained for AMP prediction, as evaluated in (Gao et al. 2024). This benchmark includes AMPs from six classic datasets, uses negative samples from UniProt, and applies CD-HIT (Fu et al. 2012) with a 40% similarity threshold to reduce homology bias. No additional fine-tuning is performed, as the BERT models are already trained specifically for AMP prediction, including classification heads. The authors also note that parts of these datasets may be present in the pretraining data, which likely benefits the BERT models on this benchmark. To mimic such pretraining with fingerprints and LightGBM, we adopt a leave-one-dataset-out strategy. For example, when evaluating on the ADAPTABLE dataset, we merge all remaining datasets into a single training set and apply CD-HIT-2D (Fu et al. 2012) at a 40% threshold to remove peptides overly similar to those in the test set.

Results are reported in Table 3, focusing on F1 score, as all models achieve nearly identical AUROC values close to 100%. Additional metrics are provided in the Supplementary Material. Fingerprint-based models achieve state-of-the-art performance on all datasets, with Topological Torsion (TT) yielding the best average F1 score. Performance differences vary by dataset but show no clear dependence on test set size or peptide size. For instance, ADAPTABLE and DRAMP both contain approximately 4000 peptides with an average length of 75 amino acids, yet differ substantially in difficulty. On ADAPTABLE, fingerprints improve F1 by 0.3% over the best BERT-based model, while on DRAMP the gain reaches 12.1%. These results demonstrate that atom-level, short-range fingerprints consistently yield strong models, but the exact data used for training has a considerable impact on the final performance.

**Table 3:** The results on BERT-based models benchmark (Gao et al. 2024).

Model	ADAPTABLE	APD	CAMP	dbAMP	DRAMP	YADAMP	Average F1
AMP-BERT	81.7	78.3	82.3	62.7	64.0	82.6	75.3
BERT-Protein	79.4	90.1	80.6	82.9	58.8	58.8	75.1
cAMPs_pred	61.3	74.1	72.8	52.4	39.2	85.0	64.1
LM_pred	55.9	71.7	64.5	60.7	47.6	82.1	63.8
LM_pred (BFD)	73.9	89.6	83.6	68.7	64.8	84.4	70.3
RDKit	**82.0**	93.0	88.4	91.4	76.4	92.0	87.2
TT	80.6	**94.3**	**88.8**	90.8	**76.9**	**95.8**	**87.8**
ECFP	80.8	93.0	88.2	**91.5**	76.3	**95.8**	87.6

#### XUAMP benchmark

Datasets used in the previous section are widely adopted in AMP research, but are relatively small. To address this limitation, a unified AMP benchmarking dataset known as XUAMP was proposed (Xu et al. 2021). It is constructed from nine commonly used datasets, such as CAMP and DRAMP, which are merged and deduplicated using homology-based CD-HIT clustering (Fu et al. 2012). This prevents data leakage by ensuring that highly similar sequences do not appear in both training and test sets, ensuring a realistic evaluation. The authors benchmarked traditional feature engineering approaches combined with various classifiers, including Random Forest, SVM, and k nearest neighbors (kNN).

We exactly replicate the original XUAMP setup and data splits, and report the results in Table 4. Fingerprint-based models again achieve SOTA performance, demonstrating robustness across different dataset sizes. The strongest prior method, AMPfun (Chung et al. 2019), relies on a relatively complex pipeline that combines binary amino acid position profiles, sequence composition features, physicochemical descriptors, and feature selection. Such pipelines can be fragile, as evidenced by the superior performance of the much simpler ECFP fingerprint in both AUROC and MCC.

**Table 4:** The results on XUAMP benchmark (Xu et al. 2021).

Method	AUROC	MCC
AMPscannerV2	58.5	0.137
iAMP-2L	59.2	0.261
ADAM-SVM	61.2	0.264
ampir	61.9	0.156
MLAMP	62.9	0.194
ADAM-HMM	68.4	0.39
AMPlify	69.7	0.381
AMPEP	72.7	0.425
AMPfun	73.5	0.414
RDKit	74.5	0.404
TT	73.1	0.385
**ECFP**	**75.0**	**0.429**

#### AMPBenchmark

The authors of AMPBenchmark (Sidorczuk et al. 2022) observed that negative class sampling has a strong impact on model performance. Their benchmark evaluates the sensitivity of AMP predictors to data choice by retraining models across a large collection of datasets with varying negative sampling strategies. Evaluated models range from sequence-based feature engineering approaches, such as AmpGram (Burdukiewicz et al. 2020), through CNN-based deep learning models like Deep-AmPEP30 (Yan et al. 2020), to multimodal ensembles combining physicochemical and sequence features, such as ampir (Fingerhut et al. 2021).

Model robustness is assessed by retraining on a suite of datasets constructed by pairing the same positive class peptides with 11 different negative sampling strategies, each repeated five times using distinct UniProt subsets. This yields 55 datasets that vary in sequence length distributions, sequence similarity, and class balance, each evaluated using an 80–20% train-test split. Results on AMPBenchmark are summarized in Table 5, reporting the mean and standard deviation across all retraining runs. Once again, fingerprint-based models achieve the best performance, with substantially lower standard deviations than competing methods. This suggests that feature engineering based on peptide topological graph yields more robust and reliable models than complex ensembles. Despite their popularity in bioinformatics, the latter are inherently susceptible to data sampling strategy and human bias in descriptor selection.

**Table 5:** The results on AMPBenchmark (Sidorczuk et al. 2022).

Model	AUROC
AmPEP	61.69 ± 6.93
iAMP-2L	62.60 ± 9.51
SVM-LZ	79.71 ± 2.43
CS-AMPPred	86.69 ± 4.36
AMAP	89.87 ± 4.18
Deep-AmPEP30	90.98 ± 5.61
AmPEPpy	95.35 ± 2.99
AmpGram	95.56 ± 2.54
MACREL	96.29 ± 2.43
MLAMP	96.68 ± 2.35
ampir	96.71 ± 2.08
AMPScannerV2	96.79 ± 2.08
TT	97.19 ± 1.89
RDKit	97.25 ± 1.84
**ECFP**	**97.37** ± **1.74**

### General peptide benchmarks

Beyond antimicrobial activity, many other peptide properties are of interest, including antioxidant activity, anti-MRSA effects, toxicity, and more. A common challenge in this area is the small size of available datasets, which often contain fewer than 1000 positive samples. To assess how fingerprint-based models perform under these constraints, we selected two large benchmarks comprising a total of 68 datasets that span a wide range of peptide properties: AutoPeptideML (Fernández-Díaz et al. 2024) and PeptideReactor (Spänig et al. 2021).

#### AutoPeptideML

AutoPeptideML (Fernández-Díaz et al. 2024) comprises 18 datasets. Positive peptides are drawn from existing literature datasets, while negative ones are carefully filtered to avoid false negatives and sampled to match the sequence length distribution of the positives. Train-test splits are created using homology clustering to produce an out-of-distribution test set, analogous to scaffold splits commonly used in molecular property prediction. Together, these choices result in a particularly challenging evaluation setup. AutoPeptideML reports results for state-of-the-art protein language models (PLMs), including models with up to 3B parameters, such as Prot-T5-XL (Elnaggar et al. 2022). Their embeddings are combined with a complex ensemble of 30 classifiers: 10 k nearest neighbors (kNN), 10 Random Forests, and 10 LightGBMs, each tuned separately via Bayesian hyperparameter optimization.

Results are summarized in Table 6. Fingerprint-based models are competitive in both MCC and AUROC with the best performing PLMs. Importantly, they are far more parameter-efficient, reaching comparable performance with roughly 22k parameters (counting tree nodes in LightGBM), compared to PLMs with up to 3 billion weights. Notably, the ECFP-based classifier outperforms three variants of ESM2 (Lin et al. 2023) in both AUROC and MCC, and achieves MCC nearly identical to ESM2-650M. A likely explanation is that peptides, unlike most proteins used to train and evaluate PLMs, are very short sequences. Fingerprints operate on full molecular graphs and can be viewed as a higher-resolution representation of peptides than sequence-only models.

**Table 6:** The results on AutoPeptideML benchmark (Fernández-Díaz et al. 2024).

Model	# params	AUROC	MCC
ProtBERT	420M	75.9	0.375
ESM2-150M	150M	77.7	0.402
Prost-T5	3B	77.1	0.409
ESM2-8M	8M	77.5	0.418
ESM2-35M	35M	78.0	0.428
ESM1b-650M	650M	78.9	0.433
ESM2-650M	650M	**79.7**	0.438
Prot-T5-XL	3B	**79.7**	**0.447**
RDKit	**20k**	76.9	0.421
TT	**23k**	77.1	0.422
ECFP	**22k**	78.1	0.437

#### PeptideReactor

PeptideReactor (Spänig et al. 2021) is a large benchmark comprising 50 datasets designed to compare peptide encoding methods. To focus on the impact of feature engineering, all models use the same Random Forest classifier with 100 trees. No classifier hyperparameter tuning or class weighting is applied, and only the feature extraction methods are tuned. The benchmark includes 48 encodings based on sequence information or structural descriptors derived from 3D structures. This setting enables a direct comparison of molecular fingerprints with a broad range of established peptide encodings from the bioinformatics literature.

For this benchmark, we perform some limited hyperparameter tuning for fingerprints. We make this exception, since authors explicitly encourage tuning for feature extraction, and perform it for many other methods, so tuning fingerprints results in more fair comparison. Further, many datasets in this benchmark contain relatively large proteins, which may benefit from slightly larger subgraphs in fingerprints. Specifically, 17 of the 50 datasets have average sequence lengths exceeding 50 amino acids, the typical upper limit for peptides, and 8 exceed 200 amino acids. See Supplementary Material for detailed statistics of all datasets. Using 5-fold cross-validation, we tune the ECFP radius in the range [2,4], the Topological Torsion path length in the range [4,6], and the RDKit path length in the range [7,9]. These remain short-range descriptors, but allow slightly larger substructures to be captured.

Since this is an encoding benchmark, we also evaluate a general “FP encoding” setting, where the fingerprint type itself is treated as a hyperparameter. This allows us to address the question *“How effective are encodings based on the topological graph (2D structure) of a molecule?”*. Such comparison is relevant because existing feature groups rely either on amino acid sequences (1D encodings) or spatial peptide structures (3D encodings). It is also a highly realistic setting, as the best encoding method is selected for each dataset. Results are summarized in Table 7. Due to space constraints, we report only the five best sequence- and structure-based encodings here, with full results provided in the Supplementary Material.

**Table 7:** The results on PeptideReactor benchmark (*Spänig* et al. 2021).

Model group	Model	Average F1
Structure	disord	53.5
	electr	60.9
	distan	61.3
	delaun	69.5
	qsar	72.8
Sequence	dde	81.3
	ngram_	81.6
	psekraac	81.8
	dist_f	82.2
	cksaap	82.4
Fingerprint	RDKit	80.6
	TT	80.6
	ECFP	81.7
	**FP encoding**	**82.9**

Fingerprints significantly outperform all structure-based encodings that rely on spatial information, despite modeling only topological molecular graphs and short-range interactions. This suggests that these smaller proteins may exhibit biological behavior that differs from that of large proteins, for which spatial folding is known to be critical. Moreover, constructing molecular graphs takes only a few seconds, whereas spatial encodings require costly tertiary structure estimation, taking approximately 85 minutes per dataset (Spänig et al. 2021).

Fingerprints are also highly competitive with the strongest sequence-based methods. Notably, the ECFP fingerprint alone ranks fourth overall in the benchmark, outperforming 45 other encodings. We also observe substantial variation in fingerprint performance across datasets. When treating the fingerprint type as a hyperparameter, the FP encoding achieves state-of-the-art performance. This indicates that fingerprints provide robust peptide representations that scale well across diverse properties and protein sizes, but the optimal choice depends on the data and task. We note that (Spänig et al. 2021) concludes that structural encodings are inferior to sequence-based ones in both computational cost and performance. Fingerprint-based encodings offer a counterexample to this conclusion, albeit relying on a topological 2D structure rather than conformational (spatial) information.

## Additional baselines

Two commonly used sequence-based baselines for proteins and peptides are amino acid counts and ESM2 (Lin et al. 2023). The former uses a bag-of-words on sequences, typically including uni- and bigram counts. ESM2 embeds a peptide by extracting amino acid embeddings and combining them with mean pooling.

Note that molecular fingerprints can be viewed as a higher-resolution alternative to those methods. They operate directly on atomic graph, rather than on sequences, which enriches the data representation, particularly in realistic low-data regimes. This structural encoding incorporates information about amino acids, such as their elemental composition and charge, capturing structural similarities between amino acids. For example, they encode the fact that asparagine and glutamine are more similar to each other than asparagine and proline. In contrast, sequence-level models must learn these relationships implicitly, often requiring more data.

We compare the two baselines to ECFP fingerprints, with results shown in Table 8. LightGBM classifier is used in all cases except PeptideReactor, where we apply Random Forest as before. We follow the same data splits and evaluation metrics as in the earlier sections. We use the ESM2-35M variant, as larger models did not improve performance, consistent with prior findings (Fernández-Díaz et al. 2024).

**Table 8:** Results of additional baselines.

Benchmark	Metric	Amino acid counts.	ESM2	ECFP
AMPBenchmark	AUROC	96.9%	97.3%	**97.4%**
AutoPeptideML	AUROC	74.6%	77.2%	**78.1%**
BERT benchmark	F1	87.5%	**89.8%**	87.6%
LRGB, Peptides-func	AUPRC	69.8%	66.0%	**74.6%**
LRGB, Peptides-struct	MAE	0.2665	0.2828	**0.2432**
PeptideReactor	F1	80.0%	76.8%	**81.7%**
XUAMP	AUROC	74.2%	**76.8%**	75.0%

Molecular fingerprints give the best results in 5 out of 7 cases. They are particularly strong on PeptideReactor, with 4.9% F1-score gain over ESM2. Further, they are always better than amino acid counts, and as such form a strong baseline for peptide property prediction.

## Sequence shuffling

To verify that short-range dependencies are sufficient, we conduct sequence shuffling experiments. A given percentage of amino acids in each training sequence is randomly permuted, acting as an adversarial perturbation. As this shuffling ratio increases, long-range interactions are progressively destroyed. If performance remains high even with fully randomized sequences, this indicates that peptide properties mainly depend on substructural composition. Additional experiments (shuffling performance for baselines, shuffling the test sequences) and detailed tables are reported in the Supplementary Material.

**Figure 4:**
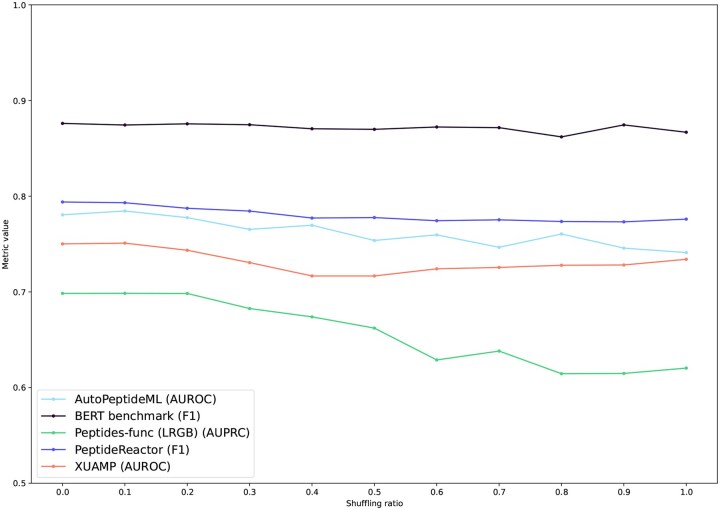
ECFP performance under increasing shuffling ratio.

We use the ECFP fingerprint with default hyperparameters and report the average metric across each benchmark (We exclude AMPBenchmark due to its very large size.) for every shuffling ratio in Figure 4. Performance degradation for fingerprint-based models is small, even with fully randomized sequences, and does not exceed about 4%. On XUAMP, results are nearly identical to those obtained without shuffling. Thus, even when sequences are completely randomized and only very short-range interactions remain, the ECFP fingerprint maintains strong performance. This further supports the hypothesis that peptide property prediction primarily depends on local, atom-level subgraphs and also demonstrates the robustness of the proposed approach.

## Additional experiments

Due to word limitations, we relegate a series of additional experiments to the Supplementary Materials. They further explore the robustness and possible limitations of the proposed approach. Here, we provide summary results.

First, we analyze error distributions and correlate them to physicochemical properties of peptides, but results do not indicate any particular patterns. Further, we explore binning data by length, e.g., testing only on shortest or longest peptides. All models show similar performance patterns, e.g., fingerprints and ESM2 underperform on shortest peptides, which have the least structural information.

Lastly, we purposefully design a long-range task, in order to delineate the limitations of the proposed method. The task is recognizing highly charged sequence motifs like “KKK” or “RRR” in sequence, which are known to impact peptide-protein binding and are highly relevant to peptide therapeutic design. It is trivial for sequence-based models like ESM2, which achieve near-perfect performance. However, it is very challenging for molecular fingerprints, as it requires using long-range and order-dependent on molecular graphs.

## Conclusions

We presented an approach to peptide function prediction based on count variants of hashed molecular fingerprints. These methods use atom-level representations derived from molecular graphs, which are inexpensive to construct, fast to compute, and do not require knowledge of the folded peptide structure. Despite relying strictly on local, short-range descriptors, they achieve strong performance across a broad range of tasks.

When combined with LightGBM, this approach delivers superior results in a large-scale evaluation spanning six benchmarks and 132 datasets. They outperform a wide range of alternatives, including GNNs, pretrained amino acid sequence transformers, hand-crafted feature engineering pipelines, and multimodal models. Beyond predictive quality, the proposed models are computationally lightweight.

To the best of our knowledge, this work provides the most extensive comparison of molecular fingerprints applied to peptides to date. In contrast to prior bioinformatics studies, we focus on count-based rather than binary fingerprints and show that this design choice is critical for strong performance. Our main methodological contribution is demonstrating that inherently short-range models achieve state-of-the-art results on peptide property prediction tasks. In particular, an ECFP-based classifier with radius 2 surpasses the previous best model on *Peptides-func* dataset from LRGB benchmark by 1.5% AUPRC. These findings challenge the presumed necessity of long-range dependencies for this task and reinforce previous observations in the literature (Tönshoff et al. 2024).

Future work will extend this approach to property prediction for larger proteins. We also plan to analyze fingerprint performance on tasks involving chemically modified peptides, such as cyclic peptides, which are of high interest for drug design and require atom-level featurization.

In conclusion, atom-level, short-range models based on molecular fingerprints constitute strong and reliable approaches for peptide property prediction. Their use is also essential for critically evaluating claims about the importance of long-range dependencies in molecular learning tasks.

## Supplementary Material

btag179_Supplementary_Data

## Data Availability

All datasets and benchmarks used are publicly available at https://github.com/scikit-fingerprints/peptides_molecular_fingerprints_classification and at https://doi.org/10.5281/zenodo.19388783.
