# Supplementary material for: Molecular fingerprints are strong models for peptide function prediction
Source: Bioinformatics. 2026 Apr 13;42(5):btag179. doi: 10.1093/bioinformatics/btag179 (PMC13143419; doi:10.1093/bioinformatics/btag179)
Supplement: btag179_Supplementary_Data [file btag179_supplementary_data.pdf]

Supplementary information

February 19, 2026

1 Reproducibility and hardware details

To ensure the full reproducibility of our results, we used `uv` to pin the exact version of all dependencies in the project, including transitive dependencies of directly used libraries. We distribute the `pyproject.toml` and resulting `uv.lock` file with our source code. This ensures the exact reproducibility of all results that is OS-agnostic and hardware-agnostic.

We conduct experiments primarily on CPU, as our method does not require GPU. Only for ESM2 we used NVidia RTX 3080 GPU with 12 GB VRAM. All experiments were run on a machine with Intel Core i7-12700KF 3.61 GHz CPU and 96 GB RAM, running Linux Ubuntu 24.04 OS. We additionally ran the experiments on a second machine with Intel Core i7-10850H 2.70 GHz CPU and 32 GB RAM, running Linux Ubuntu 22.04 OS. The results were exactly the same in all cases.

2 Targets in specific benchmarks

Here we describe in detail targets of each benchmark.

2.1 AMPBenchmark

In AMPBenchmark [11] authors focus on one task, which is antimicrobial activity. The dataset includes a test set consisting of 5 repeats and multiple training sets, each also consisting of 5 repeats. All training sets and their repeats use the same 4151 samples as their entire positive class, and all repeats of the test set use the same 1039 positive samples similarly. Repeats and training sets differ by the negatives sampling method, as described in the main body.

Each training set is defined by a sampling method used to chose negative class samples. The samples vary by number and length distribution. We report the distributions of sequence length for positive class and for negative class generated by each method in Figure 1. We also report the number of unique samples generated by each method and average number of common samples within two different repeats of the same method in Table 1.

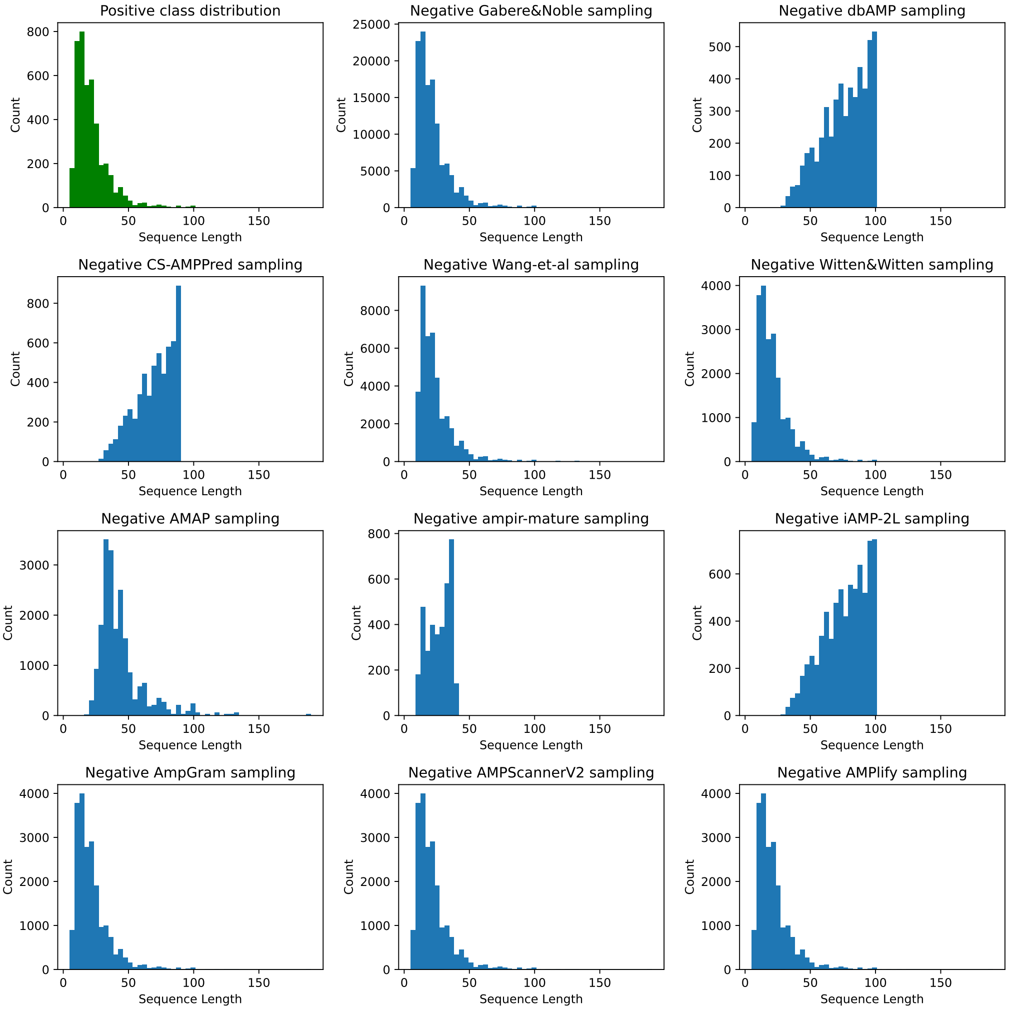

Figure 1: AMPBenchmark [11] sequence length distributions for different negative sampling methods.

Table 1: AMPBenchmark negative class counts and average common sample count off different subsampling methods.

**Sampling method**	**Unique samples**	**Avg. common samples count**
Gabere&Noble	124504	1.7
dbAMP	5138	3285.8
CS-AMPPred	5829	2942.3
Wang-et-al	41784	0.1
Witten&Witten	20754	0.1
AMAP	20046	0
ampir-mature	3579	2285
iAMP-2L	7327	4678.3
AmpGram	20754	0.1
AMPScannerV2	20755	0
AMPlify	20752	0.1

2.2 AutoPeptideML

In AutoPeptideML [4], authors use 16 independent tasks predicting targets to assess predictive performance against varied biological endpoints. Each classification task is almost perfectly balanced. These tasks include: antibacterial activity, ACE inhibition, anticancer activity, antifungal activity, antimalarial activity, antimicrobial activity, antioxidant activity, antiparasitic activity, antiviral activity, blood–brain barrier penetration, DPPIV inhibition, anti-MRSA activity, neuropeptide activity, quorum sensing activity, toxicity prediction, and tumor T-cell antigen identification.

We report sequence length distributions for each task in Figure 2. We additionally report the number of train and test samples for each of the targets in Table 2.

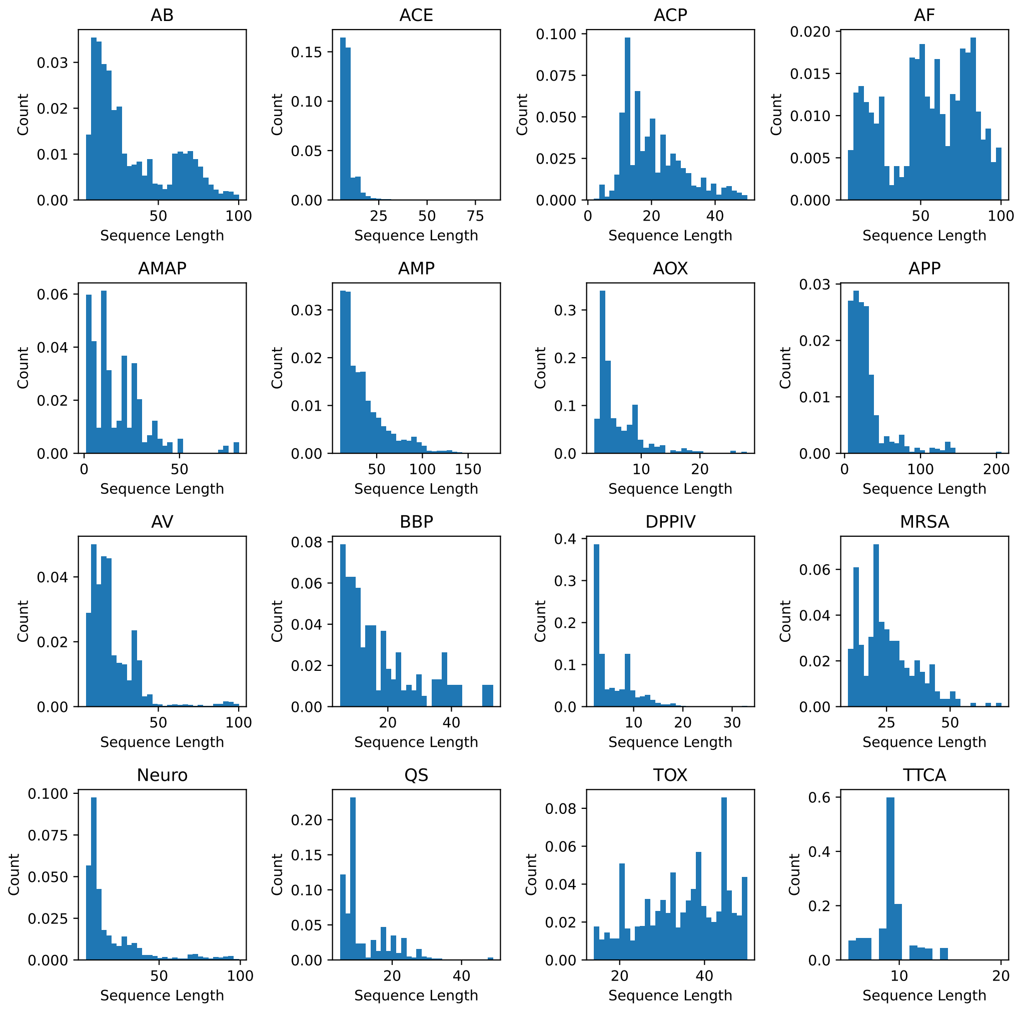

Figure 2: AutoPeptideML [4] sequence length distribution across different datasets.

Table 2: Dataset sizes in AutoPeptideML [4].

**Target**	**Train size**	**Test size**
AB	13245	3311
ACE	1685	421
ACP	1378	344
AF	1591	395
AMAP	221	55
AMP	10266	2566
AOX	698	174
APP	482	120
AV	4711	1177
BBP	191	47
DPPIV	1063	265
MRSA	237	59
Neuro	3881	969
QS	349	87
TOX	3092	772
TTCA	948	236

2.3 BERT-based models benchmark

In BERT-based models benchmark [6] authors focus on antimicrobial peptides (AMPs) classification using 6 different datasets. As described in the main body, BERT models are pretrained, and whole datasets are used for testing in the aforementioned publication. To mimic this with our approach, we use a leave-one-dataset-out strategy. For example, when evaluating on the ADAPTABLE dataset, we merge all remaining datasets into a single training set and apply CD-HIT-2D [5] at a 40% threshold to remove peptides overly similar to those in the test set.

This approach creates 6 new datasets, with the original one being the test set, and all others, after CD-HIT-2D, become the training set. We summarize the sequence length distributions of resulting datasets in Figure 3. We include the train and test set sizes, as well as percentage of positive class for each task, in Table 3. Training sets have different positive class percentage due to the applied procedure, but test sets are exactly the same, original datasets, as used in [6], and have 50% negative and positive class.

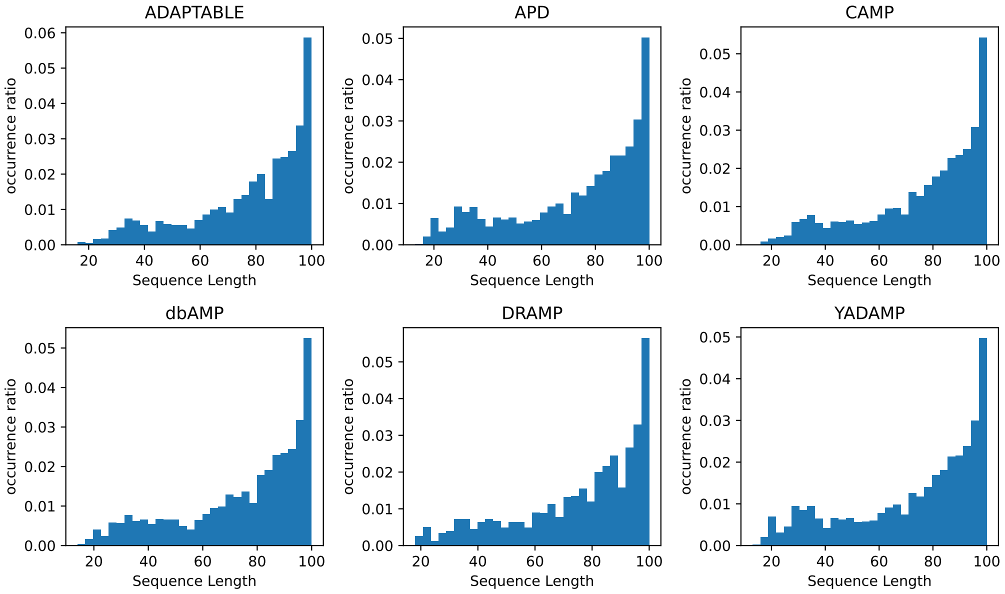

Figure 3: BERT AMP Benchmark [6] sequence length distribution across different datasets.

Table 3: Dataset sizes in BERT-based models benchmark [6].

Dataset	Train size	Train positive class %	Test size	Test positive class %
ADAPTABLE	2282	36.33%	2170	50.00%
APD	5887	49.26%	68	50.00%
CAMP	3773	43.97%	1154	50.00%
dbAMP	4905	47.46%	568	50.00%
DRAMP	2731	45.26%	2082	50.00%
YADAMP	5978	49.75%	46	50.00%

2.4 PeptideReactor

PeptideReactor [12] is a large benchmark including 50 binary classification datasets. Their tasks include A-cell epitope classification, three anticancer tasks, two antifungal tasks, two anti-inflammatory tasks, seven antimicrobial tasks, two antibacterial tasks, two antiviral tasks, linear B-cell epitope classification, eight cell-penetrating tasks, hemolytic activity classification, seventeen HIV-related tasks, immunosuppressive activity based on IL-10 induction, insect neuropeptide classification, proinflammatory activity classification, and T-cell epitope classification, all formulated as binary classification tasks.

We report sequence length distribution for each of these tasks in Figure 4. It is worth noting that many of the tasks are fixed on a specific sequence length for the entire dataset. Train-test splits are not provided for the benchmark, as it uses 10 repetitions of 5-fold cross-validation. In Table 4, we report: size of each dataset, positive class percentage, and statistics of sequence length distribution (not plots due to large number of datasets).

Table 4: Dataset sizes in PeptideReactor[12].

Dataset	Dataset size	Positive class %	Sequence length						
			**min**	**p5**	**p25**	**median**	**p75**	**p95**	**max**
ace_vaxinpad	688	44.04%	3	4	5	10	17	26	30
acp_anticp	450	50.00%	5	7	15	25	34	49	113
acp_iacp	344	40.12%	11	13	18	25	30	38	97
acp_mlacp	585	31.97%	11	13	20	25	32	45	50
afp_amppred	2768	50.00%	10	13	19	26	37	67	255
afp_antifp	2916	50.03%	4	12	41	55	73	94	100
aip_aippred	1049	40.04%	11	15	15	15	18	20	25
aip_antiinflam	2124	40.63%	7	9	15	15	19	21	30
amp_antibp	861	50.06%	30	30	30	30	30	30	30
amp_antibp2	1993	50.13%	6	13	20	27	37	53	94
amp_csamp	256	50.00%	16	29	34	47	85	110	119
amp_fernandes	231	49.78%	11	18	32	41	63	92	100
amp_gonzales	129	20.93%	11	14	20	26	38	55	84
amp_iamp2l	3284	26.77%	5	13	32	65	86	98	103
amp_modlamp	2579	47.50%	30	30	35	40	46	73	100
atb_antitbp	492	50.00%	5	7	9	12	20	38	61
atb_iantitb	492	50.00%	5	6	9	12	19	38	61
avp_amppred	1478	50.00%	10	12	17	22	34	39	255
avp_avppred	1047	57.21%	6	8	15	20	30	37	107
bce_ibce	2518	44.08%	11	12	15	15	18	22	49
cpp_cellppd	1614	50.00%	3	6	12	17	25	34	61
cpp_cellppdmod	1462	50.07%	3	7	12	16	21	30	41
cpp_cppredfl	924	50.00%	10	11	14	18	26	37	61
cpp_kelmcpp	1003	50.25%	8	12	15	19	25	30	49
cpp_mixed	128	75.78%	5	7	13	16	21	27	38
cpp_mlcpp	1903	38.78%	5	10	14	19	26	34	48
cpp_mlcppue	374	50.00%	5	7	11	15	18	27	61
cpp_sanders	145	76.55%	5	7	15	18	22	27	43
hem_hemopi	1104	47.28%	4	11	14	18	25	34	98
hiv_3tc	624	31.25%	141	240	240	241	242	245	249
hiv_abc	619	28.92%	31	240	240	241	242	245	249
hiv_apv	702	60.40%	99	99	99	99	100	102	107
hiv_azt	621	51.85%	141	240	240	241	242	245	249
hiv_bevirimat	155	27.74%	19	20	21	21	21	21	21
hiv_d4t	621	54.11%	31	240	240	241	242	245	249
hiv_ddi	623	49.12%	141	240	240	241	242	245	249
hiv_dlv	718	63.37%	170	240	240	241	242	246	253
hiv_efv	721	62.00%	74	240	240	241	242	246	253
hiv_idv	758	50.66%	99	99	99	99	100	102	107
hiv_lpv	501	44.51%	99	99	99	99	100	102	107
hiv_nfv	775	39.10%	99	99	99	99	100	102	107
hiv_nvp	733	56.62%	170	240	240	241	242	246	253
hiv_protease	947	15.73%	8	8	8	8	8	8	8
hiv_rtv	728	47.94%	99	99	99	99	100	102	107
hiv_sqv	761	60.05%	99	99	99	99	100	102	107
hiv_v3	1351	14.80%	32	34	35	35	35	35	38
isp_il10pred	1242	31.72%	8	13	15	15	15	20	42
nep_neuropipred	1750	50.00%	4	10	14	20	31	77	100
pip_pipel	3228	25.81%	11	15	15	15	15	20	25
tce_zhao	203	17.73%	10	10	10	10	10	10	10

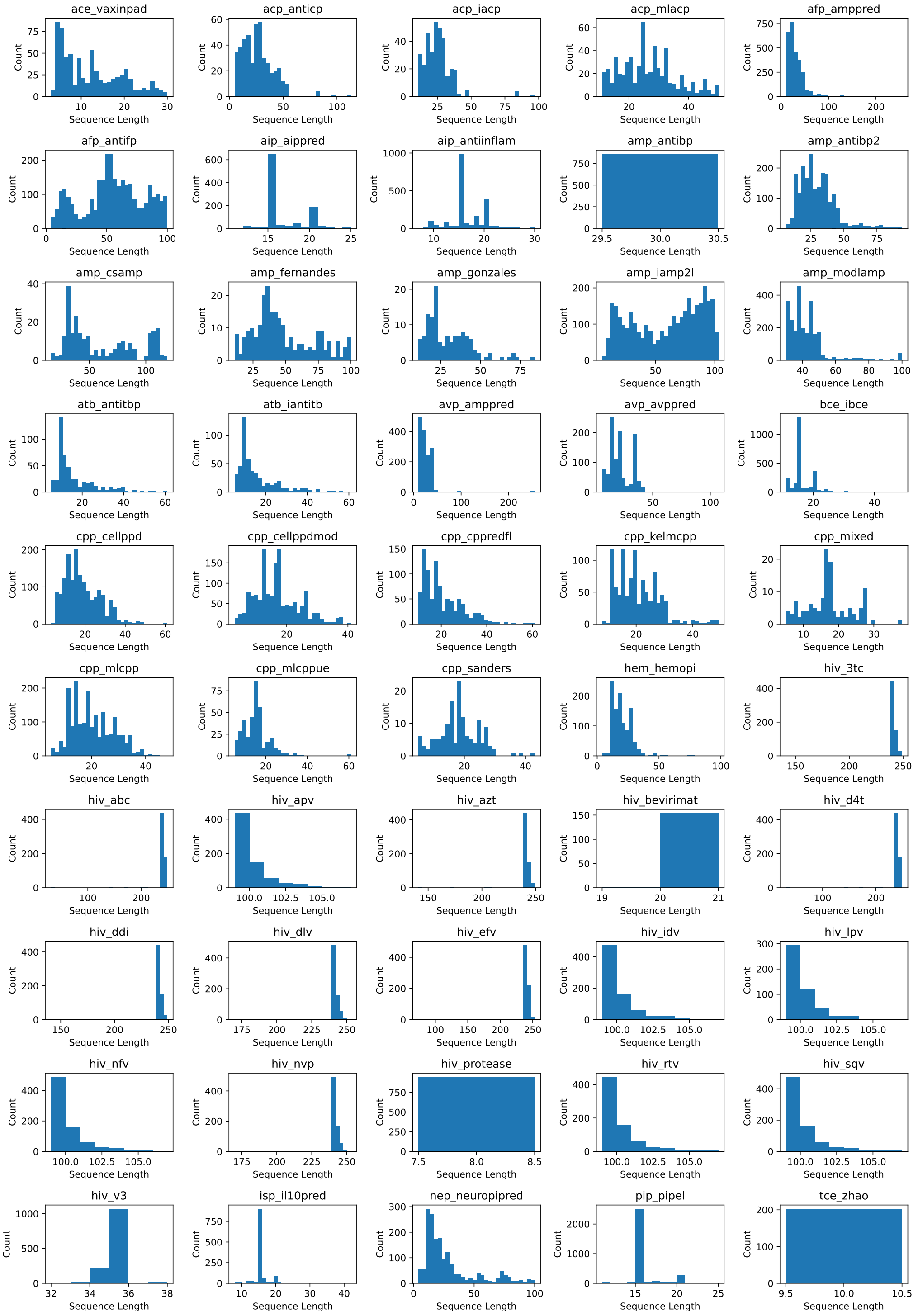

Figure 4: PeptideReactor [12] sequence length distribution across different tasks.

2.5 XUAMP

Authors of XUAMP [13] focus solely on antimicrobial peptides. The training part of this one dataset consists of 11072 samples, and testing part of 3072 samples. Both of these subsets have balanced class labels, with 50% positive and negative samples. We report the sequence length distribution from this dataset in Figure 5.

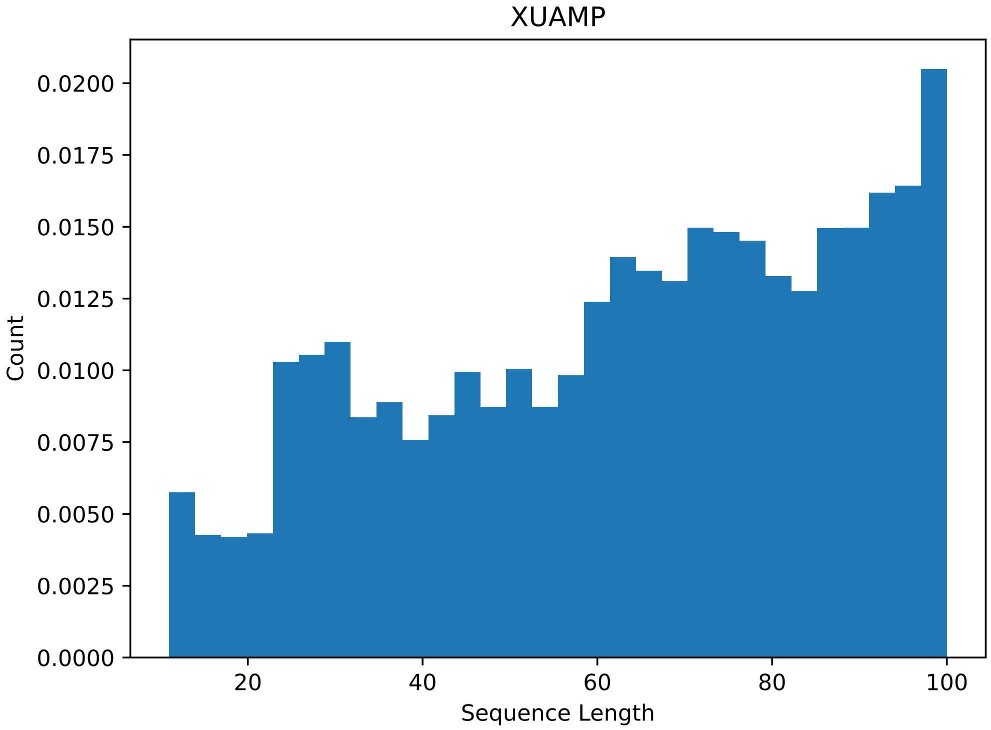

Figure 5: XUAMP [13] sequence length distribution.

2.6 LRGB

Long-Range Graph Benchmark (LRGB) [2] provides multiple datasets, of which we use Peptides-func and Peptides-struct in this work. They have the same peptides, but different labels (classification vs regression). Both datasets contain the same 13204 samples in the training and 2331 in the test set.

We note that LRGB provides valid SMILES for all peptides, but some sequences contain custom notation for chemically modified amino acids. This cannot be parsed by, e.g., ESM or amino acid counts baselines, and also cannot be used for shuffling experiments. Thus, we exclude those sequences for those cases. However, only 125 training sequences and 20 test sequences are removed this way. Based on the remaining, valid sequences we report distribution of sequence lengths as shown in Figure 6

For peptides-func the tasks include antifungal activity, cell–cell communication, anticancer activity, drug delivery vehicle functionality, antimicrobial activity, antiviral activity, antihypertensive activity, antibacterial activity, antiparasitic activity, and toxicity prediction.

For peptides-struct the targets include mass-weighted moments of inertia along principal components 1, 2, and 3, valence-weighted moments of inertia along principal components 1, 2, and 3, peptide length along the x-, y-, and z-axes, sphericity, and deviation from the best-fit plane.

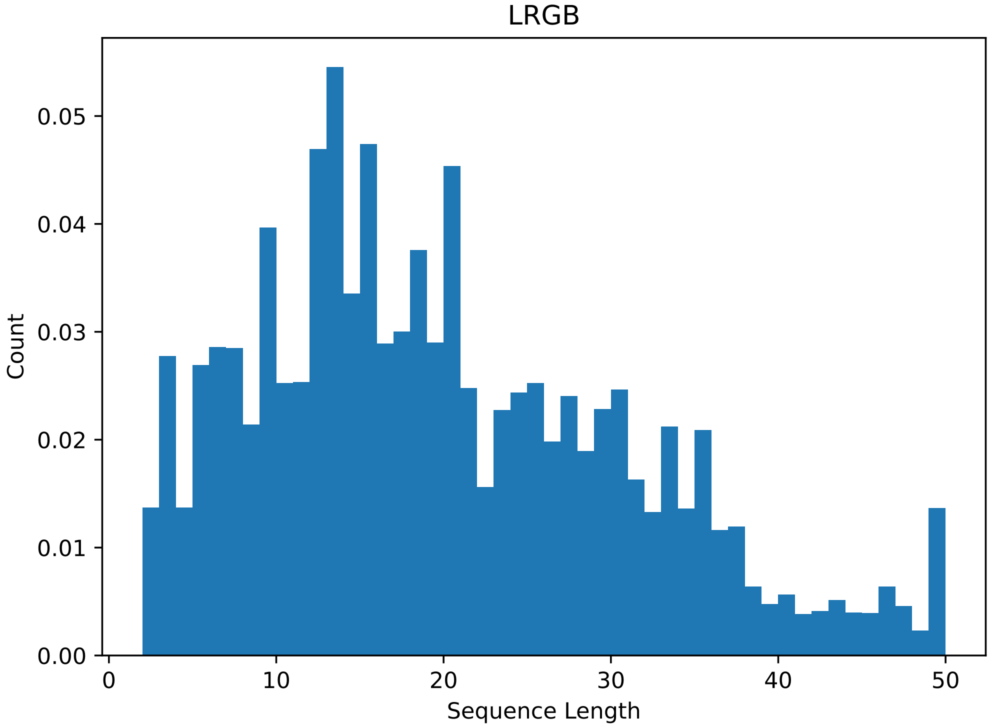

Figure 6: LRGB peptides [2] heavy atom count distribution.

3 Additional benchmark metrics

Here, we report additional metrics for AMPs benchmarks from [6] (Table 5) and [13] (Table 6), as well as AutoPeptideML benchmark [4] (Table 7). They were omitted in the main text due to space limitations.

Table 5: Results for BERT benchmark [6] results.

Metric	Model	ADAPTABLE	APD	CAMP	dbAMP	DRAMP	YADAMP	avg
**Recall**	AMP-BERT	67.60	79.40	80.40	54.20	54.70	82.60	69.82
	Bert-Protein	58.40	94.10	76.30	83.80	47.60	47.60	67.97
	cAMPs_pred	44.50	58.80	57.90	36.30	24.70	73.90	49.35
	LM_pred	40.00	55.90	49.10	45.10	31.80	69.60	48.58
	LM_pred (BFD)	64.60	88.20	78.00	58.50	52.80	82.60	59.67
	ECFP	75.60	**97.10**	88.70	**95.10**	66.80	**100.00**	87.22
	TT	75.60	**97.10**	**89.10**	94.00	**67.10**	**100.00**	87.15
	RDKit	**77.20**	**97.10**	88.70	94.00	66.50	**100.00**	**87.25**
**Precision**	AMP-BERT	80.20	77.10	84.20	74.40	77.10	82.60	79.27
	Bert-Protein	79.20	86.50	85.40	82.10	77.10	77.10	81.23
	cAMPs_pred	**98.20**	**100.00**	**97.90**	**94.50**	**94.80**	**100.00**	**97.57**
	LM_pred	92.50	**100.00**	94.00	92.80	94.00	**100.00**	95.55
	LM_pred (BFD)	86.30	91.20	90.20	83.40	83.90	86.40	91.62
	ECFP	86.90	89.20	87.70	88.20	89.00	92.00	88.83
	TT	86.40	91.70	88.50	87.80	89.80	92.00	89.37
	RDKit	87.50	89.20	88.00	89.00	89.80	85.20	88.12
**AUROC**	AMP-BERT	81.70	91.80	90.60	78.40	76.90	90.70	85.02
	Bert-Protein	79.40	96.10	90.90	91.90	74.00	74.00	84.38
	cAMPs_pred	82.30	91.80	87.00	81.90	68.10	93.00	84.02
	LM_pred	88.00	97.20	92.30	89.50	82.80	98.70	91.42
	LM_pred (BFD)	86.30	97.40	93.60	87.60	83.90	93.40	90.47
	ECFP	89.50	98.40	94.30	**97.20**	87.80	**100.00**	94.53
	TT	89.30	98.20	94.30	97.10	87.60	**100.00**	94.42
	RDKit	**90.20**	**99.30**	**94.80**	97.00	**88.20**	**100.00**	**94.92**
**F1**	AMP-BERT	81.70	78.30	82.30	62.70	64.00	82.60	75.27
	Bert-Protein	79.40	90.10	80.60	82.90	58.80	58.80	75.10
	cAMPs_pred	61.30	74.10	72.80	52.40	39.20	85.00	64.13
	LM_pred	55.90	71.70	64.50	60.70	47.60	82.10	63.75
	LM_pred (BFD)	73.90	89.60	83.60	68.70	64.80	84.40	70.32
	ECFP	80.80	93.00	88.20	**91.50**	76.30	**95.80**	87.60
	TT	80.60	**94.30**	**88.80**	90.80	**76.90**	**95.80**	**87.87**
	RDKit	**82.00**	93.00	88.40	91.40	76.40	92.00	87.20

Table 6: Additional metrics for results on [13].

**Method**	**Accuracy**	**AUROC**	**F1**	**MCC**	**Recall**	**Specificity**
AMPscannerV2	56.8	58.5	54.8	0.137	**52.3**	61.3
iAMP-2L	59.2	59.2	36.8	0.261	23.8	94.7
ADAM-SVM	61.2	61.2	47.1	0.264	34.6	87.8
ampir	56.3	61.9	37.9	0.156	26.6	85.9
MLAMP	55.3	62.9	23	0.194	13.3	97.2
ADAM-HMM	68.4	68.4	**62.3**	0.39	52.1	84.7
AMPlify	64.2	69.7	46.2	0.381	30.7	97.6
AMPEP	65.8	72.7	48.7	0.425	32.5	**99.2**
AMPfun	67.4	73.5	55.5	0.414	40.6	94.3
RDKit	67.5	73.7	58.5	0.388	45.8	89.1
ECFP	69.3	75.3	60.9	**0.426**	47.9	90.6
TT	**69.4**	**74.9**	61.7	0.424	49.3	89.5

Table 7: Additional metrics for results on AutoPeptideML [4].

**Model**	**# params**	**Accuracy**	**MCC**	**AUROC**	**F1**
ProtBERT	420M	68.5	0.375	75.9	67.7
ESM2-150M	150M	69.8	0.402	77.7	68.5
Prost-T5	3B	70.0	0.409	77.1	69.0
ESM2-8M	8M	70.2	0.418	77.5	69.4
ESM2-35M	35M	71.0	0.428	78.0	70.2
ESM1b-650M	650M	71.1	0.433	78.9	69.2
ESM2-650M	650M	68.0	0.438	**79.7**	69.7
Prot-T5-XL	3B	68.9	**0.447**	**79.7**	**70.4**
RDKit	**20k**	70.7	0.421	76.9	69.6
TT	**23k**	70.8	0.422	77.1	69.4
ECFP	**22k**	**71.5**	0.437	78.1	70.2

4 Additional classifiers on LRGB

Here, we present results of two additional classifiers on the LRGB benchmark datasets: Random Forest and Extremely Randomized Trees [7]. For both we use 500 trees, entropy or squared error as cost function (for classification and regression, respectively), and class weighting for classification. We can report standard deviations for them over 10 random seeds, because they are nondeterministic.

Peptides-func results are in Table 8, and Peptides-struct in Table 9. For clarity, we also include LightGBM results from the main body, which is a deterministic classifier and thus does not have standard deviation.

Random Forest and Extremely Randomized Trees classifiers have very low standard deviation, highlighting the stability of the fingerprint-based approach. Models based on ECFP features consistently achieve the best results in all cases, but other fingerprints and classifiers also obtain results highly competitive with long-range GNNs.

Table 8: AUPRC ↑ for different classifiers on Peptides-func.

	**LightGBM**	**Random Forest**	**Extremely Randomized Trees**
**RDKit**	73.11	71.48 ± 0.13	69.38 ± 0.06
**TT**	73.18	71.66 ± 0.09	69.86 ± 0.08
**ECFP**	74.60	73.55 ± 0.10	71.98 ± 0.08

Table 9: MAE ↓ for different classifiers on Peptides-struct.

	**LightGBM**	**Random Forest**	**Extremely Randomized Trees**
**RDKit**	0.2459	0.2459 ± 0.0002	0.2440 ± 0.0001
**TT**	0.2438	0.2471 ± 0.0003	0.2467 ± 0.0003
**ECFP**	0.2432	0.2442 ± 0.0002	0.2433 ± 0.0001

5 Additional LRGB timings

Here, we present expanded timings of fingerprints and compare them to times reported in LRGB [2] (Appendix C.2), summarizing them in Table 10. Times for fingerprint-based models are an average of 10 runs on 12-core Intel Core i7-12700KF. We measure them for *Peptides-func*, and for *Peptides-struct* results were almost identical. Timings for GNNs are taken from [2], and use Nvidia A100 GPU.

The entire time for our approach (feature extraction + classifier training) is shorter than precomputing structural embeddings or training even a single epoch of SAN model. Graph transformer model has faster epochs, they still require precomputing LapPE embeddings, while also giving much worse results than fingerprints (see main body for results table).

One should also take into consideration the difference in raw compute power (CPU vs powerful GPU) required to get those times, which makes the difference even more significant.

Table 10: Time of computation on Peptides-func.

**Model**	**Time [s]**
Transformer+LapPE	5.9
SAN+LapPE	53.6
SAN+RWSE	49.7
LapPE encoding	74
RWSE encoding	53
ECFP	19
TT	15.2
RDKit	42.8

6 Additional PeptideReactor results

Here, we present the performance of all encodings on PeptideReactor [12], in Table 11. They are sorted from highest to lowest result. Further, in Table 12, we provide results for molecular fingerprints with and without tuning their hyperparameters.

Table 11: Full results on PeptideReactor benchmark [12].

**Encoding**	**Avg F1**	**Type**
FP encoding	82.9	fingerprint
cksaap	82.4	sequence
dist_f	82.2	sequence
psekraac	81.8	sequence
ECFP	81.7	fingerprint
ngram_	81.6	sequence
dde	81.3	sequence
dpc	80.6	sequence
TT	80.6	fingerprint
RDKit	80.6	fingerprint
fldpc_	79.4	sequence
qsorde	78.9	sequence
waac_a	78.8	sequence
apaac_	78.2	sequence
aac	78.1	sequence
binary	77.9	sequence
ctdd	77.9	sequence
paac_l	77.9	sequence
aainde	77.8	sequence
tpc	76.6	sequence
cksaag	76.5	sequence
ctriad	75.9	sequence
ctdt	75.7	sequence
ctdc	75.6	sequence
ksctri	75.6	sequence
gtpc	75	sequence
gdpc	74	sequence
flgc_a	73.8	sequence
fft_aa	73.3	sequence
qsar	72.8	structure
nmbrot	72.3	sequence
zscale	72.1	sequence
eaac_w	71.6	sequence
blomap	70.9	sequence
egaac_	70.3	sequence
blosum	69.8	sequence
delaun	69.5	structure
gaac	69.5	sequence
moran_	69	sequence
socnum	69	sequence
geary_	68.9	sequence
cgr_re	68.8	sequence
distan	61.3	structure
electr	60.9	structure
disord	53.5	structure
sseb	51.7	structure
asa	51.5	structure
ta	51.4	structure
ssec	48.3	structure

Table 12: Results on PeptideReactor benchmark [12] with and without tuning.

**Fingerprint**	**Tuned?**	**F1 score**
ECFP	No	79.4%
	Yes	81.7%
Topological Torsion	No	77.8%
	Yes	80.6%
RDKit	No	80.0%
	Yes	80.6%

7 Shuffling results

Here we report additional sequence shuffling experiments. We evaluate the performance of three models for different shuffling ratios: molecular fingerprints ECFP model, amino acid counts, and ESM2. For each benchmark and model we perform two distinct experiments. In one, we shuffle only sequences from the train set, and in the other from both train and test set.

We present results for individual models in Figures 7, 8 and 9. Comparisons of those models within each benchmark are reported in Tables 13, 14, 15, 16 and 17. Results include the performance loss delta, computed as the difference between performance achieved with unshuffled and fully shuffled data.

For both variants of the experiment, our ECFP model maintains comparable performance regardless of shuffling ratio, showing only a slight decrease compared to other models. Amino acid count model performs consistently only on AutoPeptideML, BERT AMP and XUAMP. Degradation in performance of sequence-based baselines is particularly prominent for LRGB and PeptideReactor. We can see that the two baseline models perform worse in the unshuffled test set experiment. Our hypothesis is that this induces a distribution shift between shuffled train and unshuffled test sequences, resulting in quality degradation.

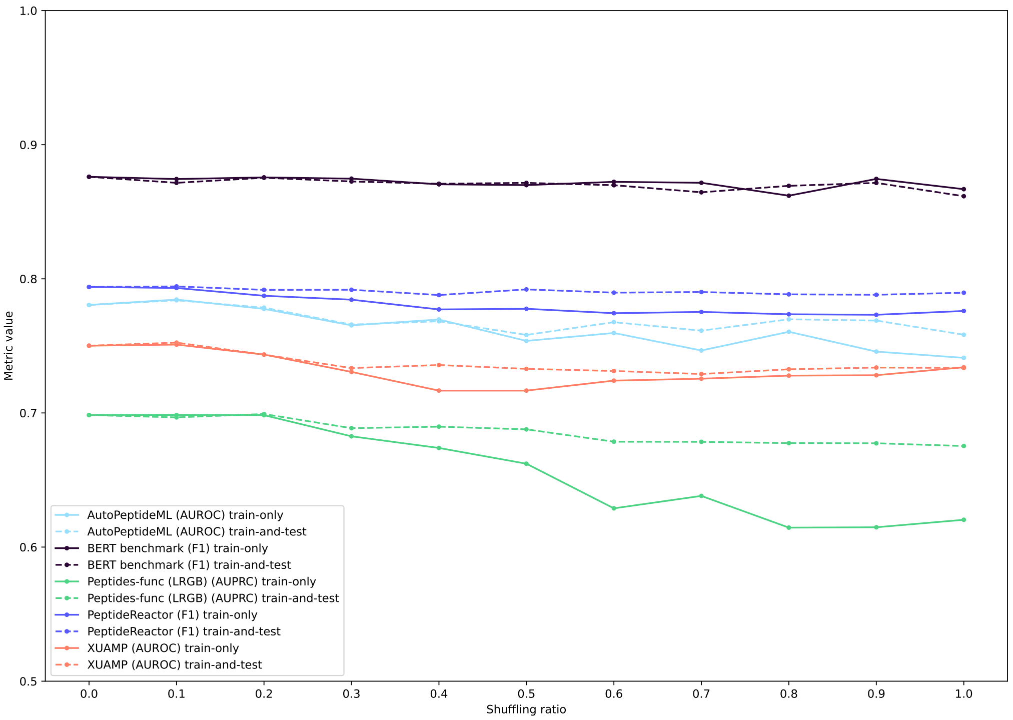

Figure 7: Shuffling metrics for ECFP + LightGBM.

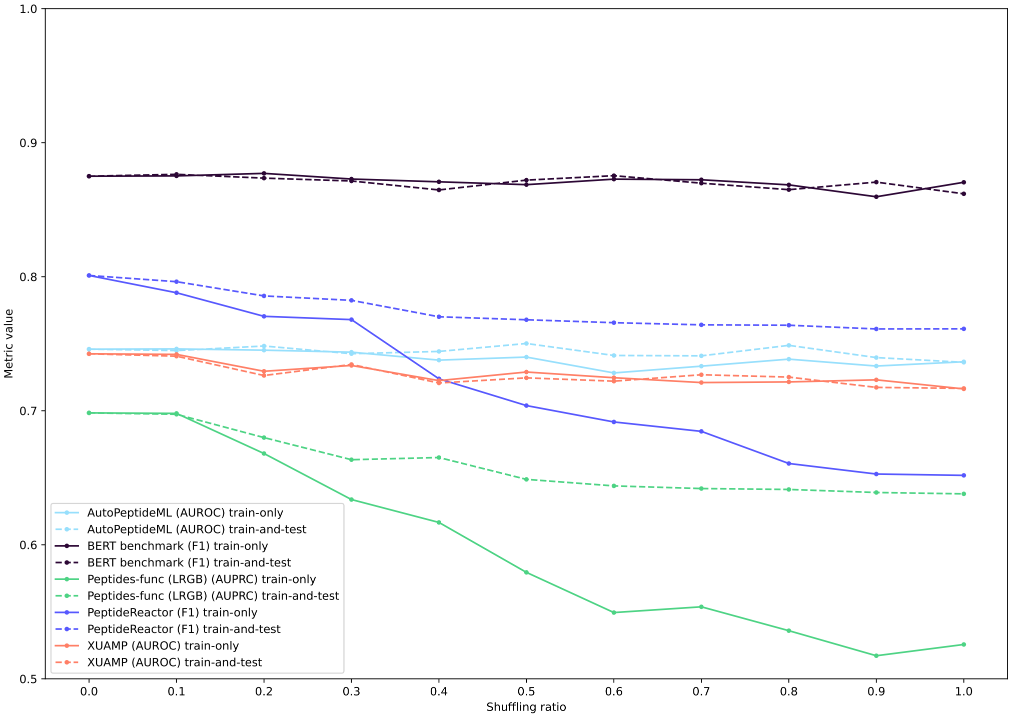

Figure 8: Shuffling metrics for amino acid counts model.

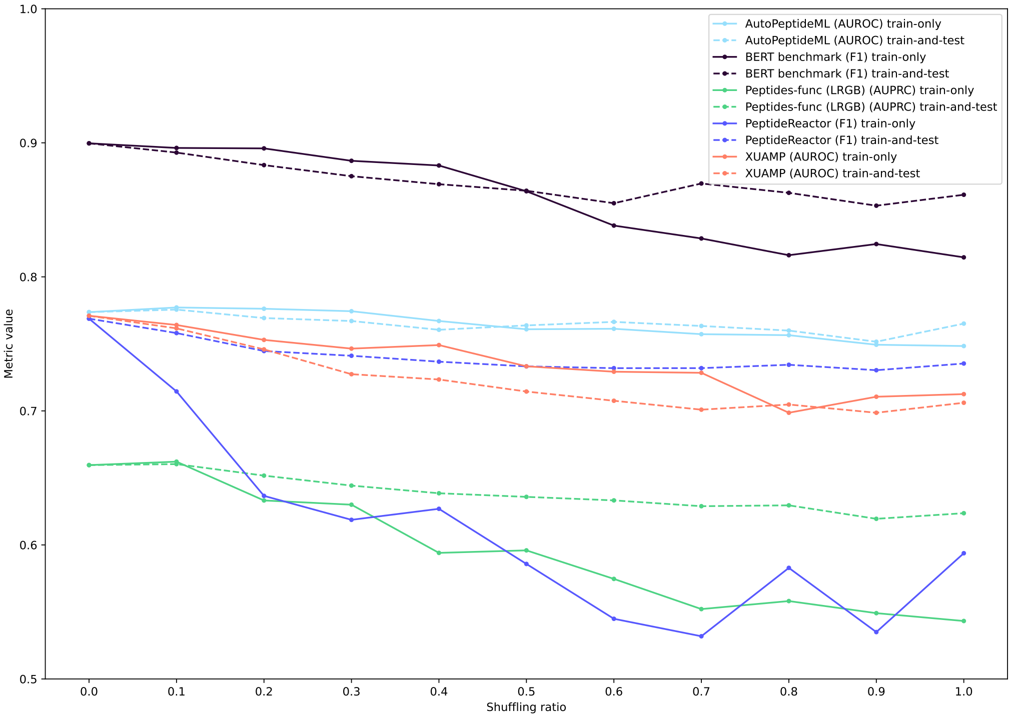

Figure 9: Shuffling metrics for ESM2 [9].

Table 13: Shuffling results for AutoPeptideML [4], AUROC metric.

Shuffle ratio	ECFP		Amino acid counts		ESM2	
	**train**	**train and test**	**train**	**train and test**	**train**	**train and test**
0.0	78.05	78.05	74.59	74.59	77.37	77.37
0.1	78.45	78.4	74.61	74.49	77.71	77.56
0.2	77.76	77.85	74.52	74.83	77.62	76.93
0.3	76.53	76.59	74.37	74.26	77.44	76.71
0.4	76.97	76.84	73.78	74.43	76.71	76.05
0.5	75.37	75.82	74.00	75.02	76.09	76.38
0.6	75.96	76.77	72.82	74.12	76.13	76.64
0.7	74.66	76.13	73.33	74.10	75.72	76.34
0.8	76.05	76.98	73.85	74.88	75.65	75.99
0.9	74.57	76.89	73.34	73.97	74.94	75.16
1.0	74.11	75.83	73.65	73.61	74.84	76.51
delta	-3.94	-2.22	-0.94	-0.98	-2.53	-0.86

Table 14: Shuffling results for BERT-based models benchmark [6], F1 metric.

Shuffle ratio	ECFP		Amino acid counts		ESM2	
	**train**	**train and test**	**train**	**train and test**	**train**	**train and test**
0.0	87.61	87.61	87.5	87.5	89.96	89.96
0.1	87.44	87.16	87.52	87.64	89.62	89.27
0.2	87.56	87.53	87.71	87.36	89.58	88.34
0.3	87.47	87.26	87.29	87.14	88.66	87.51
0.4	87.05	87.09	87.08	86.47	88.31	86.91
0.5	86.99	87.15	86.87	87.21	86.39	86.42
0.6	87.23	86.98	87.28	87.54	83.83	85.49
0.7	87.16	86.45	87.23	86.98	82.86	86.97
0.8	86.20	86.93	86.85	86.49	81.62	86.27
0.9	87.45	87.15	85.97	87.06	82.45	85.31
1.0	86.68	86.16	87.04	86.19	81.46	86.12
delta	-0.93	-1.45	-0.46	-1.31	-8.50	-3.84

Table 15: Shuffling results for LRGB Peptides-func benchmark [2], AUPRC metric.

**Shuffle ratio**	**ECFP**		**Amino acid counts**		**ESM2**	
	**train**	**train and test**	**train**	**train and test**	**train**	**train and test**
0.0	92.09	92.09	91.02	91.02	90.74	90.74
0.1	91.73	91.72	90.96	91.04	90.40	90.36
0.2	91.78	91.94	89.89	90.55	89.55	90.52
0.3	91.03	91.15	89.58	90.47	88.69	89.76
0.4	90.83	91.24	87.81	89.14	86.72	89.74
0.5	90.61	91.56	86.46	88.76	86.83	89.32
0.6	90.41	91.91	85.99	88.70	86.73	89.26
0.7	89.73	91.27	85.41	89.03	85.04	88.91
0.8	89.09	90.94	83.71	87.93	85.49	89.44
0.9	88.84	90.68	83.87	88.57	85.28	88.79
1.0	89.39	90.91	83.83	88.65	83.84	89.50
delta	-2.7	-1.18	-7.19	-2.37	-6.9	-1.24

Table 16: Shuffling results for PeptideReactor benchmark [12], F1 metric.

**Shuffle ratio**	**ECFP**		**Amino acid counts**		**ESM2**	
	**train**	**train and test**	**train**	**train and test**	**train**	**train and test**
0.0	79.39	79.39	80.09	80.09	76.88	76.88
0.1	79.32	79.43	78.80	79.63	71.45	75.81
0.2	78.74	79.18	77.04	78.57	63.66	74.47
0.3	78.44	79.18	76.80	78.24	61.87	74.11
0.4	77.72	78.79	72.38	77.01	62.69	73.67
0.5	77.77	79.21	70.39	76.79	58.58	73.32
0.6	77.44	78.97	69.16	76.57	54.49	73.19
0.7	77.53	79.02	68.46	76.41	53.18	73.19
0.8	77.36	78.84	66.06	76.38	58.29	73.44
0.9	77.32	78.81	65.28	76.10	53.50	73.04
1.0	77.60	78.96	65.18	76.11	59.38	73.53
delta	-1.79	-0.43	-14.91	-3.98	-17.50	-3.35

Table 17: Shuffling results for XUAMP benchmark [13], AUROC metric.

**Shuffle ratio**	**ECFP**		**Amino acid counts**		**ESM2**	
	**train**	**train and test**	**train**	**train and test**	**train**	**train and test**
0.0	75.01	75.01	74.25	74.25	77.10	77.10
0.1	75.1	75.24	74.2	74.07	76.41	76.16
0.2	74.35	74.35	72.94	72.63	75.30	74.59
0.3	73.06	73.34	73.38	73.45	74.65	72.74
0.4	71.66	73.57	72.25	72.07	74.91	72.34
0.5	71.66	73.29	72.89	72.45	73.33	71.44
0.6	72.41	73.13	72.46	72.21	72.92	70.76
0.7	72.55	72.9	72.10	72.69	72.84	70.09
0.8	72.78	73.25	72.15	72.51	69.86	70.48
0.9	72.81	73.38	72.31	71.74	71.06	69.86
1.0	73.41	73.35	71.63	71.67	71.25	70.61
delta	-1.60	-1.66	-2.62	-2.58	-5.85	-6.49

8 Analysis of difficult predictions and errors

To investigate whether certain data samples are systematically more difficult for the model to predict, we used the AutoPeptideML benchmark [4]. This benchmark provides multiple prediction tasks with independent datasets and challenging train-test splits.

For each dataset, we selected samples for which the absolute difference between the true class label and the predicted probability exceeded 0.9. We compared the distributions of their physicochemical properties with those of the remaining samples, with smaller error, summarized in Figure 10. Descriptors included: sequence length; counts and fractions of positively charged, negatively charged, charged, and polar amino acids, counts of cysteines, glycines, and prolines. Additionally, we use more features typical in chemoinformatics, such as molecular weight, number of heavy atoms and bonds, lipophilicity, topological polar surface area, numbers of hydrogen bond donors and acceptors, numbers of rings and number of rotatable bonds.

We use the Epps-Singleton statistical test [3] to check if there is a statistically significant difference between the two distributions for each descriptor. It was selected as we have both continuous and discrete descriptors, and this test has higher power for discrete ones than Kolmogorov-Smirnov. Because the number of high-error samples is much smaller than the remaining samples, we repeated the test 100 times using the full high-error set and bootstrap samples of the remaining data matched in size.

Out of 16 AutoPeptideML datasets (tasks), only 3 showed statistically significant differences in any feature, and no feature was consistently significant across tasks. We additionally aggregated prediction errors across the entire benchmark and repeated the analysis, which again revealed no statistically meaningful differences between the distributions of the worst predictions and the remaining samples.

Overall, these results indicate that no individual physicochemical property consistently increases prediction difficulty when using fingerprint-based representations.

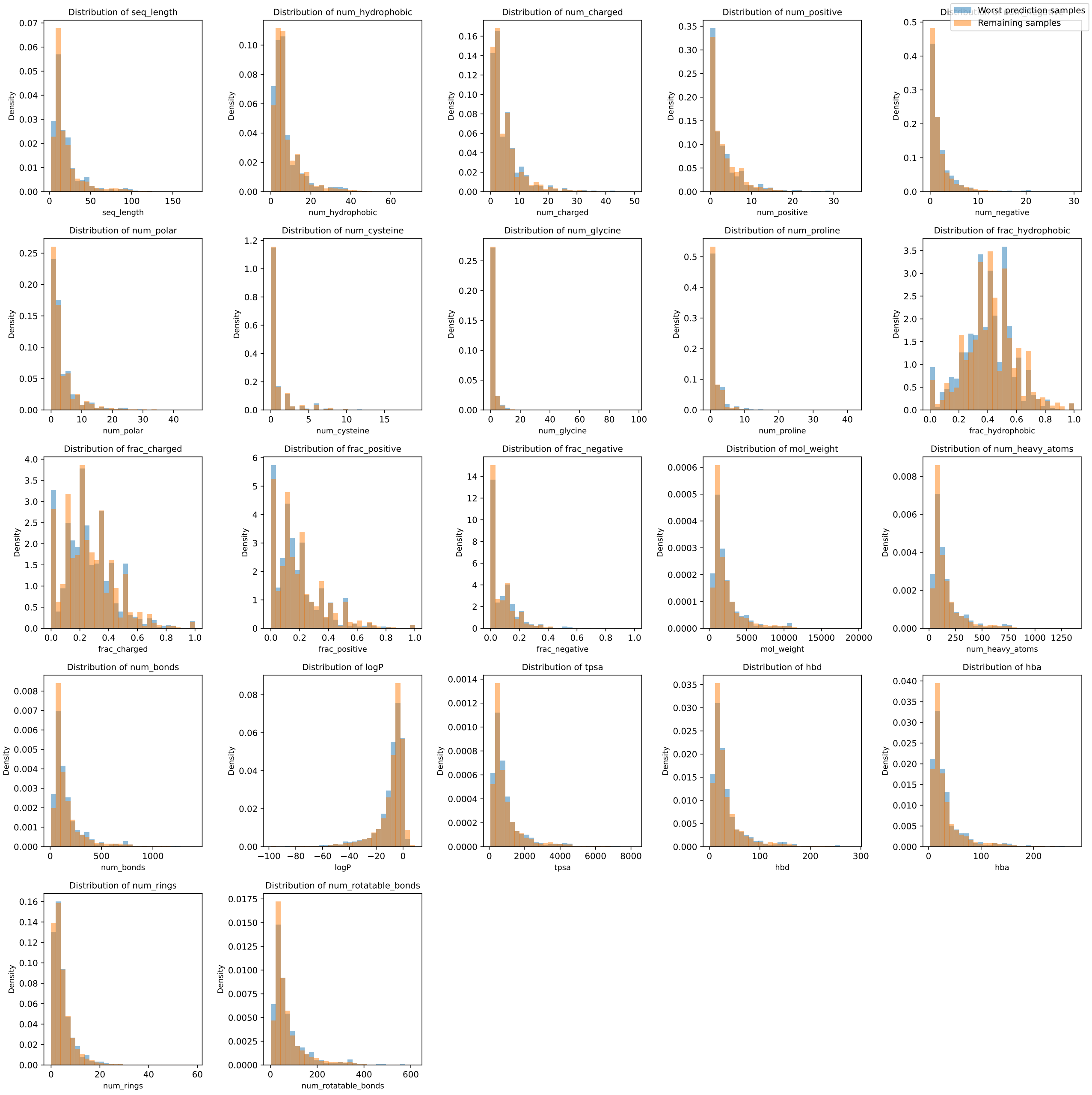

Figure 10: AutoPeptideML [4] feature distributions.

9 Sequence length split

To evaluate robustness of models to length of training and testing data, we use sequence length split. We selected 3 datasets for this experiment: AutoPeptideML, Peptides-func from LRGB, and XUAMP, and we test 3 models: ECFP, amino acid counts, and ESM2.

Peptides in each dataset are sorted by sequence length and divided into 5 length buckets: 20% of the shortest ones, those of lengths 20-40% of distribution, and so on. Each one has the same number of peptides, and we perform a cross-validation on those lengths. For example, when we test on 0-20% length sequences (i.e., 20% of shortest ones), we use all others for training. As this kind of splitting can introduce heavy class imbalance, we use MCC metric as well suited for imbalanced classification [1], and also report the average positive class percentage (averaged over datasets for AutoPeptideML).

Results are summarized in Tables 18, 19, and 20. Overall, all models exhibit quite similar behavior, with the highest results in the middle 40-60% test bucket, where they can interpolate more from the training data. Results are universally lower for shortest peptides, which contain the least information that the model can use. The performance of molecular fingerprints indicates that they are not particularly negatively affected by peptide size, performing on par with sequence-based baselines.

Table 18: Sequence length split results for AutoPeptideML benchmark.

**Model**	**0-20%**	**20-40%**	**40-60%**	**60-80%**	**80-100%**
ECFP	0.260	0.458	0.482	0.489	0.376
Amino acid counts	0.290	0.409	0.463	0.400	0.355
ESM2	0.262	0.435	0.508	0.527	0.385
Average positive class percentage	52%	51%	47%	51%	48%

Table 19: Sequence length split results for LRGB Peptides-func benchmark.

**Model**	**0-20%**	**20-40%**	**40-60%**	**60-80%**	**80-100%**
ECFP	0.149	0.368	0.418	0.321	0.215
Amino acid counts	0.230	0.419	0.452	0.393	0.268
ESM2	0.099	0.337	0.377	0.293	0.240
Average positive class percentage	13%	17%	18%	17%	17%

Table 20: Sequence length split results for XUAMP benchmark.

**Model**	**0-20%**	**20-40%**	**40-60%**	**60-80%**	**80-100%**
ECFP	0.427	0.577	0.540	0.483	0.427
Amino acid counts	0.285	0.525	0.532	0.496	0.409
ESM2	0.285	0.648	0.675	0.601	0.470
Average positive class percentage	96%	57%	42%	31%	22%

10 Sequence motif detection

There are tasks that are short-range on sequence level, yet long-range on the molecular graph level. They are challenging for the proposed molecular fingerprints approach, and we provide an example of such as task as an example of peptide therapeutic design task that benefits more from more common baselines. This delineates the applicability domain of the proposed model - if the task requires incorporating order of amino acids, particularly those far from each other in the molecular graph, the sequence-based models have a strong advantage.

The task is recognizing highly charged sequence motifs "KKK" and "RRR" in sequence, which are known to impact peptide-protein binding and are highly relevant to peptide therapeutic design [8, 10, 14]. This is basically checking if there is a substring in the amino acid sequence, and thus is trivial for PLMs like ESM2. It is much harder, but not impossible, for molecular fingerprints, as they ignore order of subgraphs, and thus have very weak learning signal in this task.

We use XUAMP dataset, with the same train and test splits, but replace the original labels with 1 if "KKK" or "RRR" is present in sequence, or 0 otherwise. About 8% of data has positive labels, so we report MCC metric, which works well for imbalanced cases [1]. Molecular fingerprints and amino acid counts use the same models as in the main body, while ESM2-35M is finetuned, in order to show its maximal possible performance. Our goal here is not to achieve any particular performance, but rather show the ability of those methods to incorporate long-range dependencies. Finetuning is kept lightweight and short, with just 2 epochs and learning rate $5 * 10^{-5}$, optimizing BCE loss.

Results are summarized in Table 21. As expected, molecular fingerprints achieve results better than random, but not great. Amino acid counts baseline performs well, while ESM2 gets a near-perfect result.

Table 21: Results on sequence motif detection task.

**Model**	**MCC**
ECFP	0.270
Topological Torsion	0.267
RDKit	0.238
Amino acid counts	0.616
ESM2	0.993

References

[1] Davide Chicco and Giuseppe Jurman. The advantages of the matthews correlation coefficient (mcc) over f1 score and accuracy in binary classification evaluation. *BMC genomics*, 21(1):6, 2020.

[2] Vijay Prakash Dwivedi, Ladislav Rampášek, Michael Galkin, Ali Parviz, Guy Wolf, Anh Tuan Luu, and Dominique Beaini. Long Range Graph Benchmark. *Advances in Neural Information Processing Systems*, 35:22326–22340, 2022.

[3] Thomas W Epps and Kenneth J Singleton. An omnibus test for the two-sample problem using the empirical characteristic function. *Journal of Statistical Computation and Simulation*, 26(3-4):177–203, 1986.

[4] Raúl Fernández-Díaz, Rodrigo Cossio-Pérez, Clement Agoni, Hoang Thanh Lam, Vanessa Lopez, and Denis C Shields. AutoPeptideML: a study on how to build more trustworthy peptide bioactivity predictors. *Bioinformatics*, 40(9):btae555, 09 2024.

[5] Limin Fu, Beifang Niu, Zhengwei Zhu, Sitao Wu, and Weizhong Li. CD-HIT: accelerated for clustering the next-generation sequencing data. *Bioinformatics*, 28(23):3150–3152, 10 2012.

[6] Wanling Gao, Jun Zhao, Jianfeng Gui, Zehan Wang, Jie Chen, and Zhenyu Yue. Comprehensive Assessment of BERT-Based Methods for Predicting Antimicrobial Peptides. *Journal of Chemical Information and Modeling*, 64(19):7772–7785, 2024. PMID: 39316765.

[7] Pierre Geurts, Damien Ernst, and Louis Wehenkel. Extremely randomized trees. *Machine Learning*, 63:3–42, 2006.

[8] Yuxiao Lai, Cao Xie, Zheng Zhang, Weiyue Lu, and Jiandong Ding. Design and synthesis of a potent peptide containing both specific and non-specific cell-adhesion motifs. *Biomaterials*, 31(18):4809–4817, 2010.

[9] Zeming Lin, Halil Akin, Roshan Rao, Brian Hie, Zhongkai Zhu, Wenting Lu, Nikita Smetanin, Robert Verkuil, Ori Kabeli, Yaniv Shmueli, Allan dos Santos Costa, Maryam Fazel-Zarandi, Tom Sercu, Salvatore Candido, and Alexander Rives. Evolutionary-scale prediction of atomic-level protein structure with a language model. *Science*, 379(6637):1123–1130, 2023.

[10] Erin E Schexnaydre, Jana Gerstmeier, Ulrike Garscha, Paul M Jordan, Oliver Werz, and Marcia E Newcomer. A 5-lipoxygenase-specific sequence motif impedes enzyme activity and confers dependence on a partner protein. *Biochimica et Biophysica Acta (BBA)-Molecular and Cell Biology of Lipids*, 1864(4):543–551, 2019.

[11] Katarzyna Sidorczuk, Przemysław Gagat, Filip Pietluch, Jakub Kała, Dominik Rafacz, Laura Bakała, Jadwiga Słowik, Rafał Kolenda, Stefan Rödiger, Legana C H W Fingerhut, Ira R Cooke, Paweł Mackiewicz, and Michał Burdukiewicz. Benchmarks in antimicrobial peptide prediction are biased due to the selection of negative data. *Briefings in Bioinformatics*, 23(5):bbac343, 08 2022.

[12] Sebastian Spänig, Siba Mohsen, Georges Hattab, Anne-Christin Hauschild, and Dominik Heider. A large-scale comparative study on peptide encodings for biomedical classification. *NAR Genomics and Bioinformatics*, 3(2):lqab039, 05 2021.

[13] Jing Xu, Fuyi Li, André Leier, Dongxu Xiang, Hsin-Hui Shen, Tatiana T Marquez Lago, Jian Li, Dong-Jun Yu, and Jiangning Song. Comprehensive assessment of machine learning-based methods for predicting antimicrobial peptides. *Briefings in Bioinformatics*, 22(5):bbab083, 03 2021.

[14] Fangyan Zhang, Ping Yang, Wenbo Mao, Chao Zhong, Jingying Zhang, Linlin Chang, Xiaoyan Wu, Hui Liu, Yun Zhang, Sanhu Gou, et al. Short, mirror-symmetric antimicrobial peptides centered on "rrr" have broad-spectrum antibacterial activity with low drug resistance and toxicity. *Acta Biomaterialia*, 154:145–167, 2022.
